# *Enterococcus faecium* and *Pediococcus acidilactici* deteriorate *Enterobacteriaceae*-induced depression and colitis in mice

**DOI:** 10.1038/s41598-022-13629-9

**Published:** 2022-06-07

**Authors:** Hyo-Min Jang, Jeon-Kyung Kim, Min-Kyung Joo, Yoon-Jung Shin, Kyung-Eon Lee, Chang Kyun Lee, Hyo-Jong Kim, Dong-Hyun Kim

**Affiliations:** 1 Neurobiota Research Center, College of Pharmacy, Kyung Hee University, 26, Kyungheedae-ro, Dongdaemun-gu, Seoul, 02447 Korea; 2 College of Pharmacy, Jeonbuk National University, 26, Jeonju, 54896 Korea; 3 Department of Internal Medicine, Kyung Hee University School of Medicine, Seoul, 02447 Korea

**Keywords:** Immunology, Microbiology

## Abstract

Gut dysbiosis is closely associated with the outbreak of inflammatory bowel disease (IBD) and psychiatric disorder. The *Enterobacteriaceae* population was higher in the feces of patients with inflammatory bowel disease (IBD-F) than in those of healthy control volunteers (HC-F). The *Enterococcaceae* and *Lactobacillaceae* populations were higher in the feces of IBD patients with depression (IBD/D^+^-F) vs. the feces of IBD patients without depression (IBD/D^−^-F). Therefore, we examined the effects of *Klebsiella oxytoca, Escherichia coli, Cronobacter sakazakii, Enterococcus faecium,* and *Pediococcus acidolactici* overpopulated in IBD/D^+^-F and their byproducts LPS and exopolysaccharide (EPS) on the occurrence of depression and colitis in mice. Oral gavages of *Klebsiella oxytoca, Escherichia coli,* and *Cronobacter sakazakii* belonging to *Enterobacteriaceae,* singly or together, caused dose-dependently colitis and depression-like behaviors in germ-free and specific-pathogen-free mice. Although *Enterococcus faecium* and *Pediococcus acidolactici* did not significantly cause colitis and depression-like behaviors, they significantly deteriorated *Klebsiella oxytoca*- or *Escherichia coli*-induced colitis, neuroinflammation, and anxiety/depression-like behaviors and increased blood LPS, corticosterone, and IL-6 levels. The EPSs from *Enterococcus faecium* and *Pediococcus acidolactici* also worsened *Klebsiella oxytoca* LPS-induced colitis, neuroinflammation, and depression-like behaviors in mice and increased the translocation of fluorescein isothiocyanate-conjugated LPS into the hippocampus. However, *Bifidobacterium longum,* which was lower in IBD/D^+^-F vs. IBD/D^−^-F, or its EPS suppressed them. In conclusion, *Enterococcus faecium* and *Pediococcus acidolactici,* known as a probiotic strain, and their EPSs may be a risk factor for the outbreak of depression and IBD.

## Introduction

Inflammatory bowel disease (IBD), including ulcerative colitis (UC) and Crohn’s disease (CD), is a chronic and recurrent disorders characterized by alternately repeating exacerbations and remissions^1^. Although obscure, the aetiology of IBD is thought to be the dysregulation of the mucosal immune system in the gut, resulting in an abnormal inflammatory response to environmental factors such as gut microbiota^2,3^. In patients with IBD, the prevalence of psychiatric disorders such as anxiety and depression is significantly higher than it is in healthy individuals^4,5^. Evidence in support of the close connection between IBD and psychiatric disorders stems primarily from animal and human studies^5,6^. Exposure to an acute psychological stress increases the secretion of the pro-inflammatory cytokines tumor necrosis factor (TNF)-α, interleukin (IL)-1β, and IL-6 in both the blood and colon mucosa of patients with IBD^7,8^. In the Manitoba IBD Cohort Study population, 80% of patients with both IBD and anxiety disorder received the diagnosis of anxiety disorder more than 2 years before the IBD diagnosis^9^. Excessive exposure of mice to stressors, such as immobilization and pathogens, stimulates the secretion of adrenal hormones, such as cortisol, and immune cytokines, such as IL-1β and IL-6, via the activation of the hypothalamus − pituitary − adrenal (HPA) axis, resulting in the occurrence of colitis and gut dysbiosis accompanied by anxiety, depression, and memory impairment^8,10–12^. Antidepressant drugs attenuate colitis and anti-inflammatory drugs alleviate psychiatric disorders with colitis^13,14^. These findings suggest that the brain can bidirectionally communicate with the gut through the HPA and gut − brain axes.

The gut microbiota of healthy humans and animals consist of bacteria, viruses, fungi, archaea, and multicellular parasites^15^. Of these, commensal opportunistic pathogens, such as *Escherichia coli* and *Klebsiella oxytoca*, produce toxic byproducts, including lipopolysaccharide (LPS)^16,17^. Their overgrowth by stressors, such as antibiotics and immobilization stress, dysregulate gut immune homeostasis, resulting in gut inflammation through the excessive expression of proinflammatory cytokines^8,11^. The chronic stimulation of proinflammatory cytokines attenuates the expression of tight-junction proteins in the intestine, resulting in a leaky gut, as observed in patients with IBD^18,19^. The induction of leaky gut by gut inflammation accelerates the absorption of bacterial byproducts, such as LPS, into the blood and alters the gut microbiota composition, which is termed dysbiosis^18,20,21^. Patients with IBD exhibit a reduced gut microbial diversity with the increased Proteobacteria populations compared with healthy individuals^22–24^. The fecal microbiota transplantation (FMT) from patients with IBD causes the colitis in transplanted germ-free mice^25,26^. The FMT from mice with colitis also causes anxiety/depression with colitis in transplanted specific-pathogen-free mice^10,11^. The fecal microbiota of patients with inflammatory bowel disease patient feces (IBD-F) have the higher abundance of *Enterobacteriaceae* population and LPS levels compared to those of healthy control volunteers (HC-F)^25^. *Enterococcaceae* and *Lactobacillaceae* populations were higher in the feces of IBD patients with depression (IBD/D^+^-F) vs. the feces of IBD patients without depression (IBD/D^−^-F)^25^. The occurrence of colitis and psychiatric disorders triggered by intestinal environmental factors, such as antibiotics and pathogens, can be attenuated by treatment with commensal Lactobacilli and Bifidobacteria^11,27^. These findings suggest that the microbiota can communicate with the brain through the microbiota − gut − brain (MGB) axis, and that the interaction between gut microbiota may control the occurrence of gut inflammation and neuropsychiatric disorders.

Therefore, to understand the role of gut bacteria in the occurrence of IBD and depression, we examined the effects of gut bacteria overpopulated in IBD-F and their byproducts LPS and exopolysaccharide (EPS) on the occurrence of anxiety/depression and colitis in mice.

## Results

### Gut bacteria overpopulated in patients with IBD/D^+^ or IBD/D^−^

**Figure 1:**
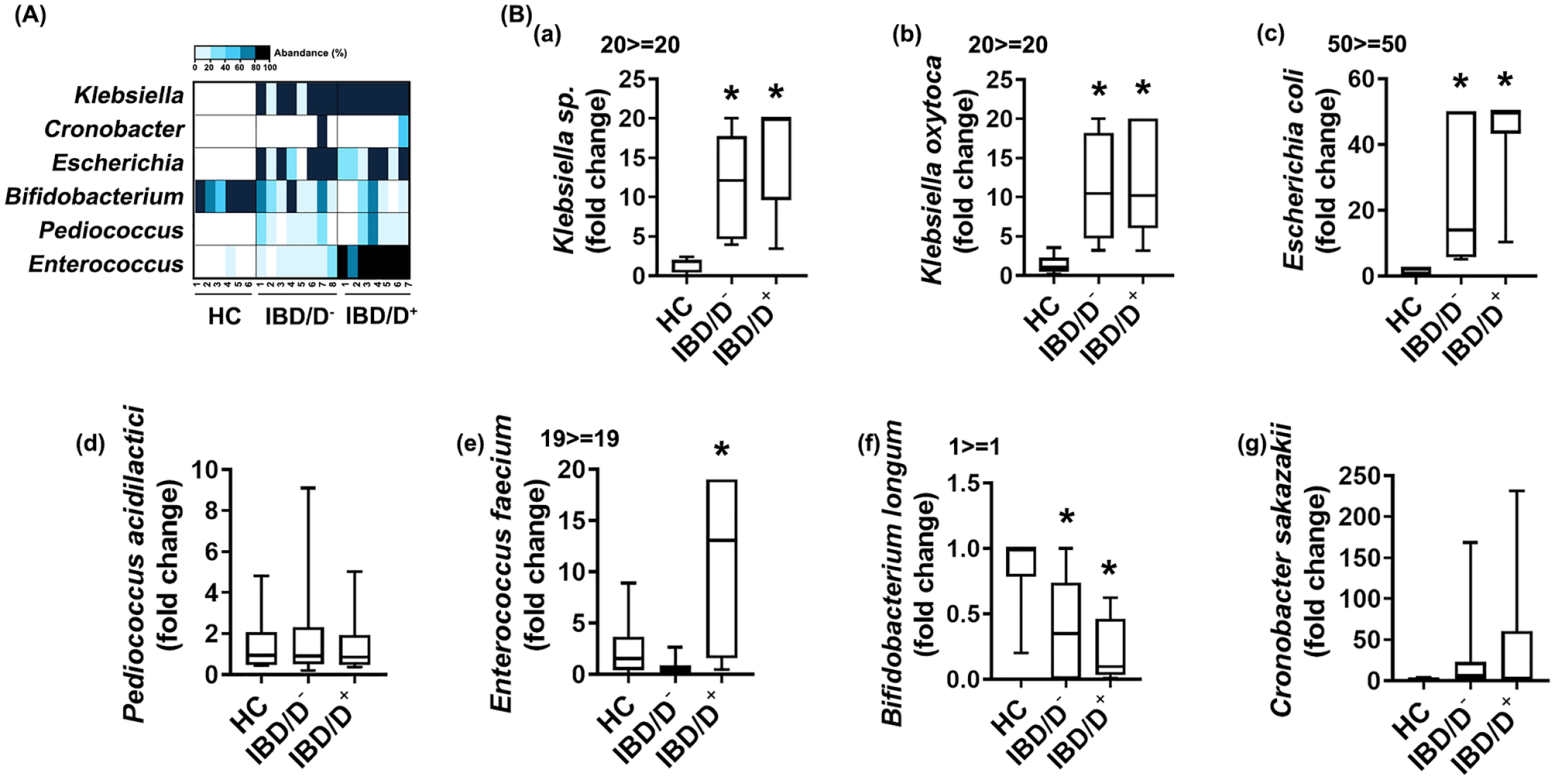
The fecal microbiota composition of patients with IBD/D^−^ or IBD/D^+^ and healthy individuals (HCs). (**A**) Effects on the levels of gut bacteria *Klebsiella sp.*, *Cronobacter sp.,* and *Escherichia sp.* grown in the DHL agar plate, *Bifidobacterium* sp. in the BL agar plate, and *Pediococcus* sp. and *Enterococcus* sp. in the mEn agar plate. (**B**) Effects on the levels of gut bacteria *Klebsiella sp.* (a), *Klebsiella oxytoca* (b), *Escherichia coli* (c), *Pediococcus acidilactici* (d), *Enterococcus faecium* (e), *Bifidobacterium longum* (f) and *Cronobacter sakazekii* (g), assessed by qPCR. Data are shown as box plots (HC n = 6; IBD/D^−^ n = 8; IBD/D^+^, n = 7). Means with same letters are not significantly different (p < 0.05). (b,c), Kruskal–Wallis test (nonparametric test).

First, we analysed the live gut bacteria composition of HC-F, IBD/D^–^F, and IBD/D^+^-F using *Enterobacteriaceae*-selective DHL, *Lactobacillaceae*-selective MRS and *Enterococcaceae*-selective mEn, and *Bifidobacteriaceae*-selective BL agar plates (Fig. 1A). *Klebsiella* sp. (including *Klebsiella oxytoca*), *Escherichia coli* and *Cronobacter sakazakii* populations (in DHL agar plates) and *Enterococcus* sp*.* (including *Enterococcus faecium*) and *Pediococcus acidilactici* populations (in mEn and MRS agar plates) were highly detected in the IBD-F compared with HC-F. The *Enterococcus* sp. and *Klebseilla* sp. populations were the highest in the IBD/D^+^-F, followed by IBD/D^−^-F and HC-F, However, the *Bifdiobacterium* sp. population including *Bifidobacterium longum* was the highest in the HC-F, followed by the feces of patients with IBD/D^−^ and IBD/D^+^. To confirm whether *Enterobacteriaceae*, *Enterococcaceae*, and *Lactobacillaceae* were associated with the occurrence of IBD and/or depression, we selected *Klebsiella oxytoca* and *Escherichia coli,* belonging to *Enterobacteriaceae*, *Enterococcus faecium* belonging to *Enterococcaceae,* and *Pediococcus acidilactici belonging to Lactobacillaceae,* which were highly detected in IBD/D^+^-F, and analyzed their populations in the HC-F and IBD-F by using qPCR (Fig. 1B). Their populations were more highly detected in the IBD-F compared with HC-F, while the *Bifidobacterium longum* population was lower in the IBD/D^+^-F vs. HC-F. The population of *Enterococcus faecium* was higher in the IBD/D^+^-F vs. IBD/D^–^F. However, *Enterococcus faecium, Pedicoccus acidilactici,* and *Bifidobacterium longum* did not exhibit hemolytic and gelatinolytic activities, like a well-known probiotics, while *Enterococcus faecium* was resistant to multi-antibiotics including ampicillin and streptomycin and produced biofilm (data not shown).

### Effects of IBD/D^+^-F-abundant *Enterobacteriaceae, Enterococcaceae, and Lactobacillaceae* on the occurrence of colitis and anxiety/depression in mice

**Figure 2:**
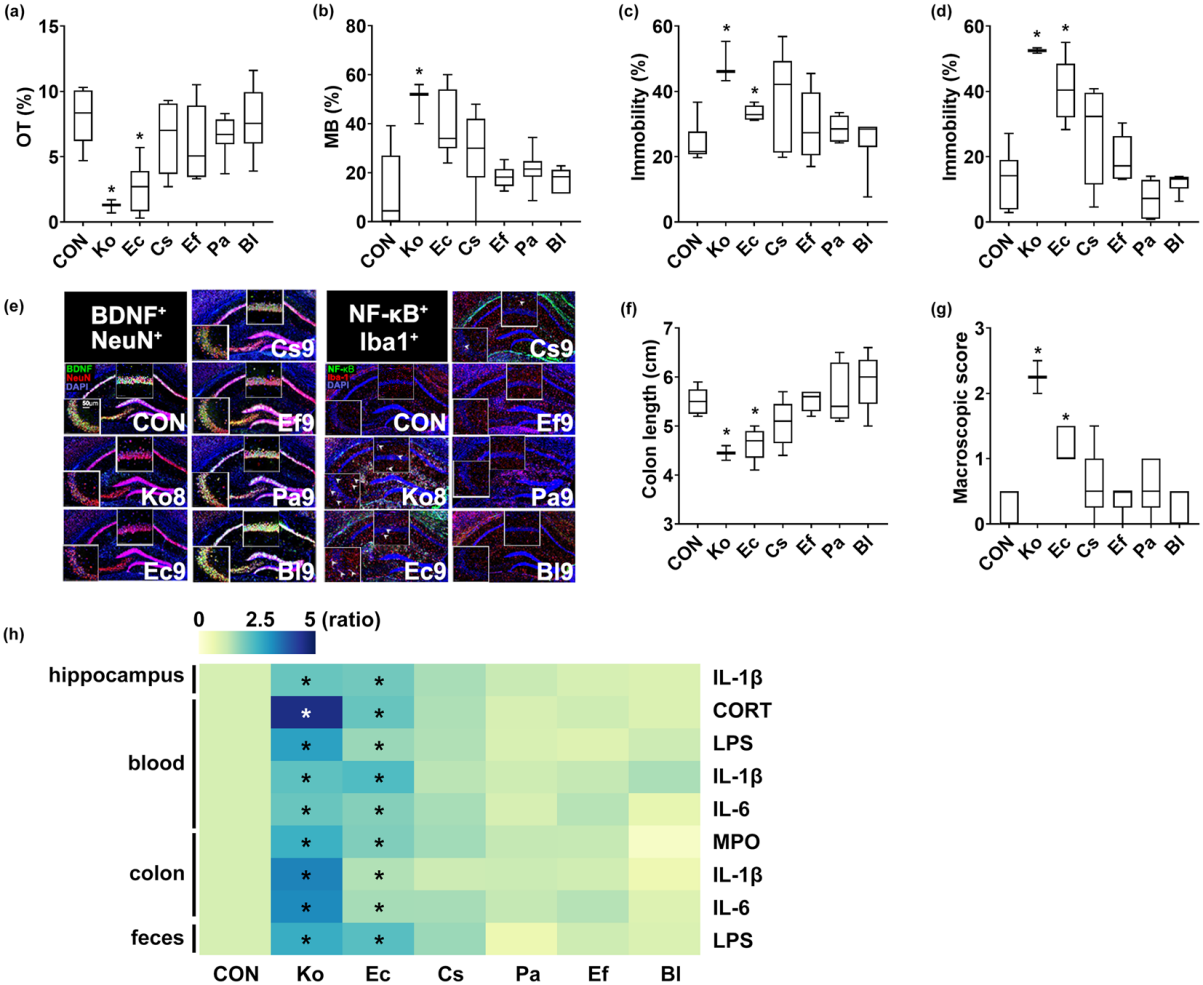
Effect of gut bacteria on the occurrence of anxiety/depression and colitis in mice. Effects on the time spent in open arms (OT) in the EPMT (**a**), marble-buried number in MBT (**b**), immobility time in the TST (**c**), and forced FST (**d**), and effects on the BDNF^+^/NeuN^+^ and NF-κB^+^/Iba1^+^ cell population in the hippocampus (**e**). Effects on the colon length (**f**) and macroscopic score (**g**). (**h**) Effects on the IL-1β, IL-6, LPS, myeloperoxidase (MPO), and corticosterone levels, indicated as compared to control group (CON: 1). Heatmap was generated using Plotly (https://chart-studio.plotly.com). *Klebsiella oxytoca* (Ko)*, Escherichia coli* (Ec), *Cronobacter sakazakii* (Cs), *Enterococcus faecium* (Ef)*, Pediococcus acidilactici* (Pa), or *Bifidobacterium longum* (Bl) at a dose of 1 × 10^9^ CFU/mouse/day was orally gavaged in mice once a day for 5 days. Control mice (Con) were treated with vehicle (saline) instead of the gut bacterial suspension. Data are shown as box plots (n = 6). *p < 0.05 vs Con. Means with same letters are not significantly different (p < 0.05). All were analyzed using unpaired *t* test.

To understand the interactive effects of IBD/D^+^-F-abundant gut bacteria on the occurrence of colitis and anxiety/depression, we isolated *Klebsiella oxytoca, Escherichia coli, Cronobacter sakazakii, Enterococcus faecium, Pediococcus acidilactici* and *Bifidobacterium longum* and examined their effects on the occurrence of colitis and anxiety/depression in specific-pathogen-free mice (Fig. 2). Of these bacteria, *Klebsiella oxytoca* exhibited the highest lethal toxicity in specific-pathogen-free mice, followed by *Escherichia coli*. Oral gavage of *Klebsiella oxytoca* at doses of 1 × 10^8^ and 1 × 10^9^ CFU/mouse/day was lethal in 0% and 50% of mice within 5 days, respectively (Supplementary Figs. S1 and S2). Oral gavage of *Escherichia coli, Cronobacter sakazakii, Enterococcus faecium, Pediococcus acidilactici* or *Bifidobacterium longum* at a dose of 1 × 10^9^ CFU/mouse/day did not kill any mice. *Klebsiella oxytoca* at doses of ≥ 1 × 10^7^ CFU/mouse/day or *Escherichia coli* at doses of ≥ 1 × 10^8^ CFU/mouse/day caused anxiety-like behavior in the elevated plus maze task (EPMT) and marble burying task (MBT) (Fig. 2a,b, Supplementary Figs. S1 and S2). Oral gavage of *Klebsiella oxytoca* at doses of ≥ 1 × 10^7^ CFU/mouse/day, *Escherichia coli* at doses of 1 × 10^9^ CFU/mouse/day caused depression-like behaviors in the tail suspension test (TST), and forced swimming test (FST) (Fig. 2c,d, Supplementary Figs. S1 and S2). Furthermore, their treatments increased the IL-1β expression, and the NF-κB^+^/Iba1^+^, LPS^+^/Iba1^+^, and IL-1R^+^ cell populations in the hippocampus and LPS levels in the blood while the BDNF^+^/NeuN^+^ cell population and claudin-5 expression decreased (Fig. 2e,h, Supplementary Figs. S1 and S2). However, oral gavage of *Cronobacter sakazakii* at a dose of 1 × 10^9^ CFU/mouse/day did not cause anxiety-/depression-like behaviors.

*Klebsiella oxytoca* at doses of ≥ 1 × 10^7^ CFU/mouse/day or *Escherichia coli* at doses of ≥ 1 × 10^8^ CFU/mouse/day also caused colitis in mice: they increased the stenosis score, myeloperoxidase activity, IL-1β and IL-6 expression, and the NF-κB^+^/CD11c^+^ cell population in the colon while the claudin-1 expression was decreased (Fig. 2f–h, Supplementary Figs. S1 and S2). They also increased corticosterone, IL-1β, IL-6, and LPS levels in the blood, as well as LPS level in the feces. However, *Cronobacter sakazakii* at a dose of 1 × 10^9^ CFU/mouse/day weakly, but not significantly, caused colitis in mice.

**Figure 3:**
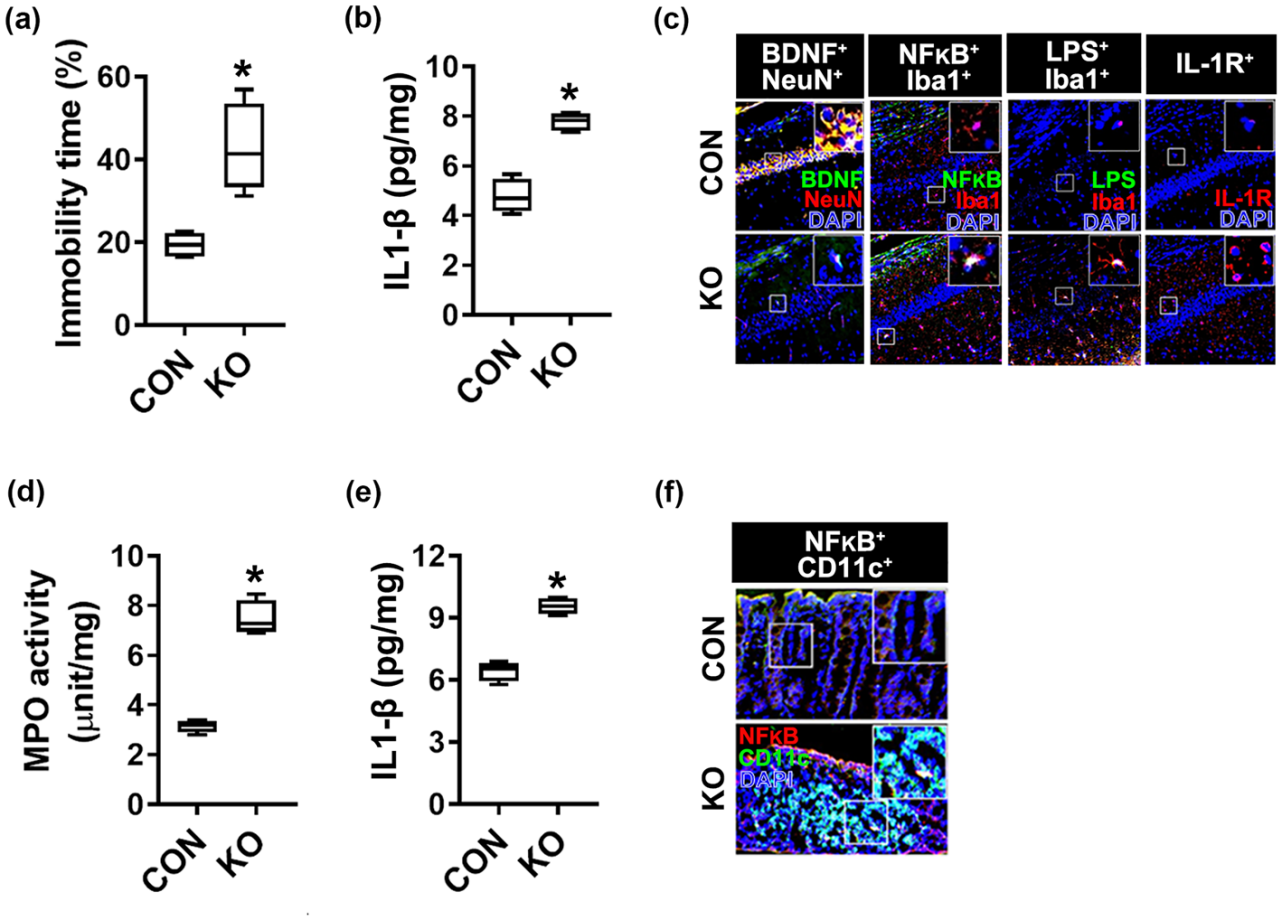
Effect of *Klebsiella oxytoca* on the occurrence of depression and colitis in germ-free mice. Effect on the occurrence of depression-like behaviors (**a**) and hippocampal IL-1β level (**b**), BDNF^+^/NeuN^+^ (**c**), NF-κB^+^/Iba1^+^ (**d**), LPS^+^/Iba1^+^ (**e**), and IL-1R^+^ cell populations (**f**) in germ-free mice. *Klebsiella oxytoca* (KO, 1 × 10^7^ CFU/mouse/day) were orally gavaged for 5 days in mice (n = 6, in specific-germ-free mice; n = 4, in germ-free mice). Control mice (CON) were treated with vehicle (saline) instead of the bacterial suspension. Data are shown as box plots. Means with same letters are not significantly different (p < 0.05). All were analyzed using unpaired t-test.

To understand whether gut microbiota-induced colitis and depression in between germ-free mice and specific-pathogen-free mice were different, we orally gavaged *Klebsiella oxytoca* into germ-free mice and examined its effects on the occurrence of colitis and depression in specific germ-free mice (Fig. 3, Supplementary Fig. S3). Oral gavage of *Klebsiella oxytoca* at a dose of 1 × 10^7^ CFU/mouse/day caused colitis and anxiety/depression in germ-free mice. *Klebsiella oxytoca* caused dose-dependently depression-like behavior, increased NF-κB^+^/Iba1^+^ and LPS^+^/Iba1^+^ cell populations, upregulated IL-1β expression, and decreased the BDNF^+^/NeuN^+^ cell population in the hippocampus. *Klebsiella oxytoca* also upregulated IL-1β expression, myeloperoxidase activity, and the NF-κB^+^/CD11c^+^ cell population in the colon. The incidence of colitis and depression in germ-free mice by *Klebsiella oxytoca* at a dose of 1 × 10^7^ CFU/mouse/day was similar to one treated at a dose of 1 × 10^8^ CFU/mouse/day in specific-pathogen-free mice.

However, oral gavage of *Enterococcus faecium, Pediococcus acidilactici,* or *Bifidobacterium longum* at a dose of 1 × 10^9^ CFU/mouse/day did not significantly cause colitis and anxiety-/depression-like behaviors in mice.

### Interactive effects of *Klebsiella oxytoca, Escherichia coli,* and *Cronobacter sakazakii* on the occurrence of depression and colitis in mice

**Figure 4:**
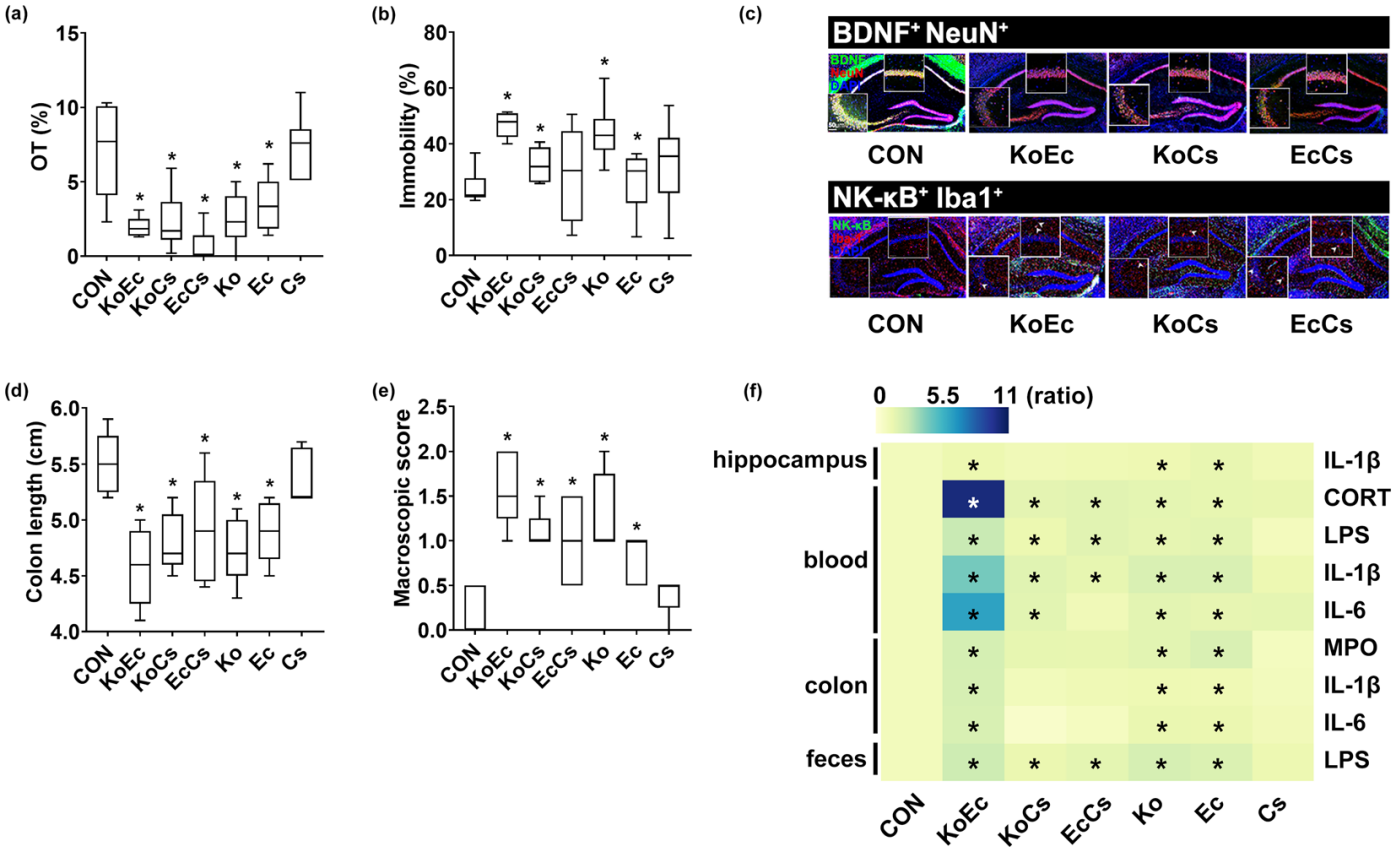
Combined effects of two bacteria belonging to *Enterobacteriaceae* on the occurrence of anxiety/depression and colitis in mice. Effects on the occurrence of anxiety/depression in the EPMT (**a**) and TST (**b**). Effects on the BDNF^+^/NeuN^+^ and NF-κB^+^/Iba1^+^ cell population in the hippocampus (**c**). Effects on the colon length (**d**) and macroscopic score (**e**). (**f**) Effects on the IL-1β, IL-6, LPS, MPO, and corticosterone levels, indicated as compared to control group (CON: 1). Heatmap was generated using Plotly (https://chart-studio.plotly.com). Among *Klebsiella oxytoca* (Ko), *Escherichia coli* (Ec), and *Cronobacter sakazakii* (Cs), two bacterial (KoEc, 1:1 of KO and Ec; KoCs, 1:1 of Ko and Cs; EcCS, 1:1 of Ec and Cs) combinations at a dose of 1 × 10^8^ CFU/mouse/day were orally gavaged once a day for 5 days in mice. Control mice were treated with vehicle (saline) instead of gut bacterial suspension. Data are shown as box plots (n = 6). *p < 0.05 vs Con. All were analyzed using unpaired *t* test.

To understand the interactive effects of gut bacteria belonging to *Enterobacteriaceae* on the occurrence of colitis and depression, we examined the combined effects of two bacteria among *Klebsiella oxytoca, Escherichia coli,* and *Cronobacter sakazakii* (Fig. 4)*.* These combinations additively increased the mortality and the occurrence of depression and colitis in mice. Of these combinations, those of *Klebsiella oxytoca* with *Escherichia coli* (KoEc, 1:1) and *Klebsiella oxytoca* with *Cronobacter sakazakii* (KoCs, 1:1) at a dose of 1 × 10^9^ CFU/mouse killed 50% of mice (Supplementary Figs. S4 and S5). The combination of *Escherichia coli* with *Cronobacter sakazakii* (1:1) at a dose of 1 × 10^9^ CFU/mouse did not kill mice. KoEc, KoCs, and EcCs caused anxiety-/depression-like behaviors in the EPMT and TST (Fig. 4a,b). They also increased IL-1β expression, and the NF-κB^+^/Iba1^+^, LPS^+^/Iba1^+^, and IL-1R^+^ cell populations in the hippocampus while the BDNF^+^/NeuN^+^ cell population and claudin-5 expression were decreased (Fig. 4c, Supplementary Figs. S4 and S5). They also increased corticosterone, IL-1β, IL-6, and LPS levels in the blood (Fig. 4f). KoEc also caused colitis most strongly, followed by KoCs and EcCs (Fig. 4d–f, Supplementary Figs. S4 and S5). They also increased LPS level in the feces.

### Interactive effects of *Enterococcus faecium*, *Pediococcus acidilactici,* and *Bifidobcterium longum* on the occurrence of colitis and anxiety/depression by *Enterobacteriaceae*

**Figure 5:**
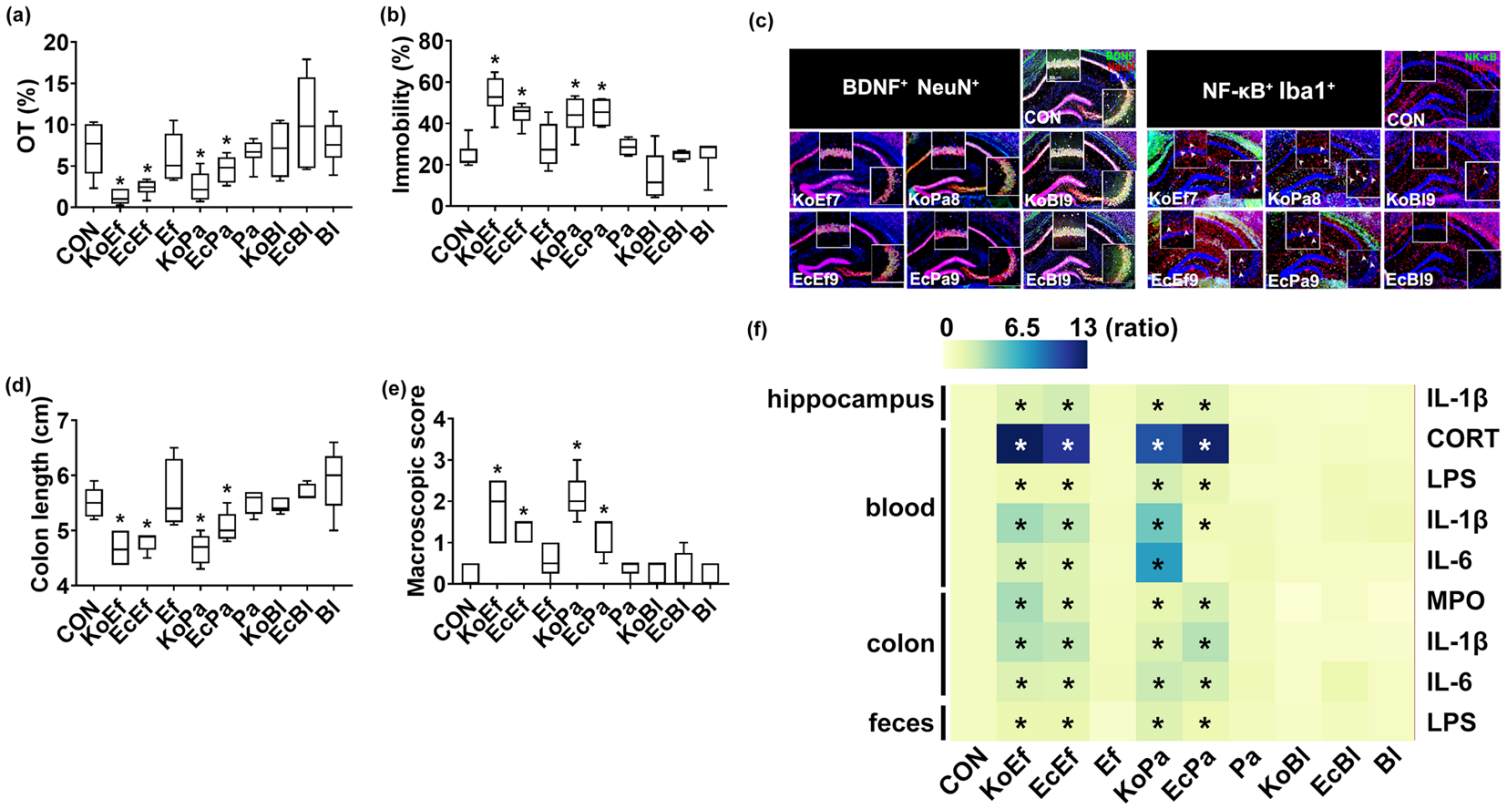
Combined effects of *Enterococcus faecium* (Ef)*, Pediococcus acidilactici* (Pa), or *Bifidobacterium longum* (Bl) with *Enterobacteriaceae* on the occurrence of anxiety/depression and colitis in mice. Effects on the occurrence of anxiety/depression in the EPMT (**a**) and TST (**b**). Effects on the BDNF^+^/NeuN^+^ and NF-κB^+^/Iba1^+^ cell population in the hippocampus (**c**). Effects on the colon length (**d**) and macroscopic score (**e**). (**f**) Effects on the IL-1β, IL-6, LPS, MPO, and corticosterone levels, indicated as compared to control group (CON: 1). Heatmap was generated using Plotly (https://chart-studio.plotly.com). Each two bacteria (KoEf, 1:1 of Ko and Ef; KoPa, 1:1 of Ko and Pa; KoBl, 1:1 of Ko and Bl; EcEf, 1:1 of Ec and Ef; EcPa, 1:1 of Ec and Pa; EcBl, 1:1 of Ec and Bl) combination at a dose of 1 × 10^7^ (KoEf), 1 × 10^8^ (KoPa), or 1 × 10^9^ CFU/mouse/day (EcEf, EcPa, EcBl, Ef, Pa, and Bl) was orally gavaged once a day for 5 days in mice. Control mice were treated with vehicle (saline) instead of gut bacterial suspension. Data are shown as box plots (n = 8). *p < 0.05 vs Con. All were analyzed using unpaired *t* test.

We examined the combined effects of *Enterococcus faecium, Pediococcus acidilactici,* and *Bifdiobacterium longum* on the occurrence of colitis and depression by *Klebsiella oxytoca* and *Escherichia coli* in mice (Fig. 5, Supplementary Figs. S6 and S7). Among the tested combinations, that of *Klebsiella oxytoca* with *Pediococcus acidilactici* (KoPa, 1;1) or *Enterococcus faecium* (KoEf, 1;1) at a dose of 1 × 10^9^ CFU/mouse/day killed 100% of mice (Supplementary Fig. S4). These combinations at doses of ≤ 1 × 10^8^ CFU/mouse/day did not kill any mice. Oral gavage of *Klebsiella oxytoca* with *Pediococcus acidilactici* (KoPa, 1;1) or *Enterococcus faecium* (KoEf, 1:1) caused anxiety-/depression-like behavior in the EPMT and TST (Fig. 5a,b). The combined treatment with *Escherichia coli* with *Pediococcus acidilactici* (EcPa, 1:1) or *Enterococcus faecium* (EcEf, 1:1) also caused anxiety-/depression-like behaviors. They also increased the NF-κB^+^/Iba1^+^, LPS^+^/Iba1^+^, and IL-1R^+^ cell populations and IL-1β expression in the hippocampus while the BDNF^+^/NeuN^+^ cell population and claudin-5 expression were decreased (Fig. 5c, Supplementary Figs. S6 and S7). Furthermore, they increased corticosterone, IL-1β, IL-6, and LPS levels in the blood (Fig. 5f, Supplementary Figs. S6 and S7). However, oral gavage of *Bifidobacterium longum* significantly protected the occurrence of anxiety/depression by *Klebsiella oxytoca* or *Escherichia coli.*

Oral gavage of *Enterococcus faecium* or *Pediococcus acidilactici* also increased the occurrence of colitis by *Klebsiella oxytoca* or *Escherichia coli* (Fig. 5d-f, Supplementary Figs. S6 and S7)*.* They increased colon shortening, myeloperoxidase activity, stenosis score, IL-1β and IL-6 expression, and NF-κB^+^/CD11c^+^ cell populations in the colon and LPS level in the feces, while the claudin-1 expression decreased. However, oral gavage of *Bifidobacterium longum* significantly protected the occurrence of colitis by *Klebsiella oxytoca* or *Escherichia coli*.

### Effects of EPSs isolated from* Enterococcus faecium, Pediococcus acidilactici,* and* Bifidobacterium longum* on the occurrence of colitis and depression by LPS isolated from *Klebsiella oxytoca*

**Figure 6:**
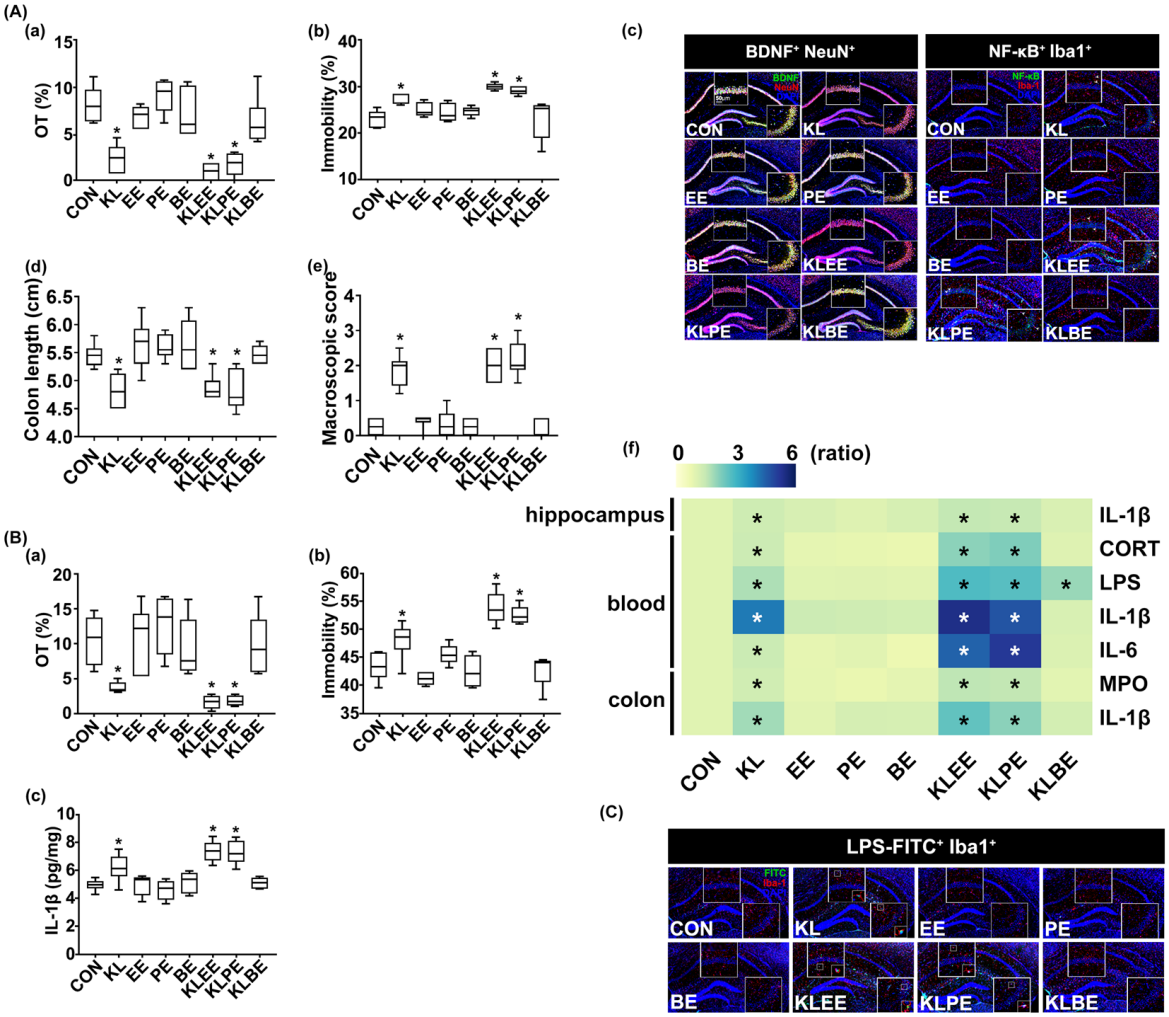
Effects of gut bacteria EPS and LPS on the occurrence of anxiety/depression and colitis in mice. (**A**) Effects of orally gavaged EPS and/or LPS. Effects on the occurrence of anxiety/depression in the EPMT (a) and TST (b). Effects on the BDNF^+^/NeuN^+^ and NF-κB^+^/Iba1^+^ cell population in the hippocampus (c). Effects on colon length (d) and macroscopic score (e). (f) Effects on the IL-1β, IL-6, LPS, MPO, and corticosterone levels, indicated as compared to control group (CON: 1). Heatmap was generated using Plotly (https://chart-studio.plotly.com). (**B**) Effects of intraperitoneally injected EPS and/or LPS on the occurrence of anxiety/depression in the EPMT (a) and TST (b) and expression of IL-1β in the hippocampus (c). (**C**) Effects of EPSs on the translocation of FITC-conjugated LPS into the brain. Test agents (CON, vehicle [saline]; EE, 20 μg/kg (i.p.) or 20 mg/kg (p.o.) of *Enterococcus faecium* exopolysaccharide; PE, 20 μg/kg (i.p.) or 20 mg/kg (p.o.) of *Pediococcus acidilactici* exopolysaccharide; BE, 20 μg/kg (i.p.) or 20 mg/kg (p.o.) of *Bifidobacterium longum* exopolysaccharide; KL, 20 μg/kg (i.p.) or 20 mg/kg (p.o.) of *Klebsiella oxytoca* lipopolysaccharide*;* KLEE, 20 μg/kg (i.p.) or 20 mg/kg (p.o.) of Ko and Ef (1:1); KLPE, 20 μg/kg (i.p.) or 20 mg/kg (p.o.) of Ko and Pa (1:1); KLBE, 20 μg/kg (i.p.) or 20 mg/kg (p.o.) of Ko and Bl (1:1) were treated in mice once a day for 5 days. FITC-conjugated LPS was orally gavagaed daily for 2 days from 24 h after EPS or LPS conjugated without FITC was gavaged for 3 days. Control mice were treated with vehicle (saline) instead of gut bacterial suspension. Data are shown as box plots (n = 8). *p < 0.05 vs Con. All were analyzed using unpaired *t* test.

To understand the synergistically accelerative component(s) of *Enterococcus faecium* and *Pediococcus acidilactici* on the occurrence of anxiety/depression by *Enterobacteriaceae* anxiety/depression, we isolated EPSs and cytosolic fraction (CF) from *Enterococcus faecium, Pediococcus acidilactici*, and *Bifidobacterium longum* and LPS from *Klebsiella oxytoca* and examined the effects of EPSs and CFs on the occurrence of anxiety/depression in mice by LPS (Fig. 6, Supplementary Fig. S8). Oral gavage of EPSs or CFs did not cause anxiety-/depression-like behaviors and colitis, while that of *Klebsiella oxytoca* LPS significantly caused anxiety-/depression-like behaviors and colitis (Fig. 6A). The combined oral gavage of *Enterococcus faecium* EPS or *Pediococcus acidilactici* EPS with *Klebsiella oxytoca* LPS increased the occurrence of anxiety/depression than that of *Klebsiella oxytoca* LPS alone, while their CFs were not affect them (data not shown). Furthermore, EPSs increased LPS-induced NF-κB^+^/Iba1^+^ cell population and IL-1β and IL-6 expression and reduced LPS-suppressed BDNF^+^/NeuN^+^ cell population and claudin-5 expression in the hippocampus. They also synergistically increased LPS levels in the blood and myeloperoxidase activity and IL-1β expression in the colon, while the claudin-1 expression decreased in the colon. However, *Bifidobacterium longum* EPS significantly suppressed LPS-induced anxiety-/depression-like behaviors, hippocampal NF-κB^+^/Iba1^+^ cell population, and blood LPS level and induced LPS-suppressed hippocampal BDNF^+^/NeuN^+^ cell population and claudin-5 expression. Intraperitoneal injections of EPSs weakly, but not significantly, caused anxiety/depression and neuroinflammation, while that of LPS significantly caused anxiety-/depression-like behaviors and neuroinflammation (Fig. 6B). However, the combined injection of *Enterococcus faecium* EPS or *Pediococcus acidilactici* EPS with *Klebsiella oxytoca* LPS increased the occurrence of anxiety/depression more strongly than that of *Klebsiella oxytoca* LPS alone. However, *Bifidobacterium longum* EPS significantly suppressed the occurrence of anxiety/depression.

To confirm the accelerative effects of EPSs on the occurrence of anxiety/depression by *Klebsiella oxytoca* LPS, we orally gavaged fluorescein isothiocyanate (FITC)-conjugated LPS (fLPS) in mice (Fig. 6C, Supplementary Fig. S8). In the hippocampus of fLPS-treated mice, fLPS was detected. Furthermore, the simultaneous treatments of fLPS with EPSs increased fLPS amount and fLPS^+^/Iba1^+^ cell population in the hippocampus more than one without EPSs. However, *Bifidobacterium longum* EPS significantly reduced the fLPS^+^/Iba1^+^ cell population in the hippocampus.

**Figure 7:**
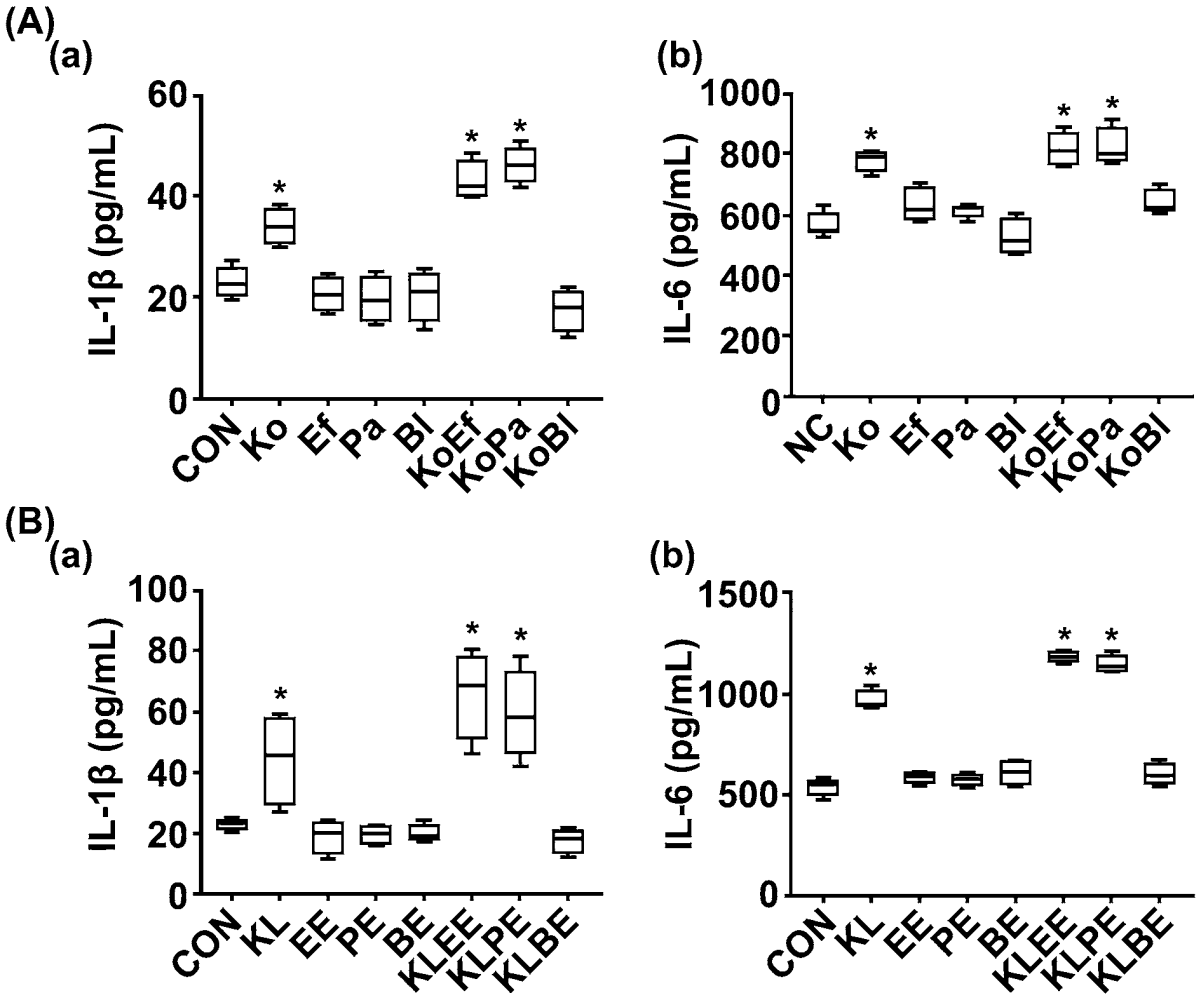
Effects of *Enterococcus faecium, Pediococcus acidilactici*, and their exopolysaccharides on *Klebsiella oxytoca*- or its lipopolysaccharide-induced IL-1β and IL-6 expression and NF-κB activation in macrophages. (**A**) Effects of gut bacteria on IL-1β (a) and IL-6 expression (b) in macrophages. (**B**) Effects of gut bacterial LPS and EPS on IL-1β (a) and IL-6 expression (b) in macrophages. Test agents (CON, vehicle [saline]; Ef, 1 × 10^5^ CFU/mL of *Enterococcus faecium* (Ef); Pa, 1 × 10^5^ CFU/mL of *Pediococcus acidilactici*; Bl, 1 × 10^5^ CFU/mL of *Bifidobacterium longum*; Ko, 1 × 10^5^ CFU/mL of *Klebsiella oxytoca;* KoEf, 1 × 105 CFU/mL of Ko and Ef (1:1); KoPa, 1 × 10^5^ CFU/mL of Ko and Pa (1:1); KoBl, 1 × 10^5^ CFU/mL of Ko and Bl (1:1). KL, 100 ng/mL of Ko lipopolysaccharide (LPS); EE, 100 ng/mL of Ef expolysaccharide (EPS); PE, 100 ng/mL of Pa; BE, 100 ng/mL of Bl; KLEE, 100 ng/mL of Ef EPS with 100 ng/mL of Ko LPS; KLPE, 100 ng/mL of Pa EPS with 100 ng/mL of Ko LPS; KLBE, 100 ng/mL of Bl EPS with 100 ng/mL of Ko LPS) was treated in macrophages. The macrophages were treated with LPS (80 ng/mL) in the presence or absence of Ef, Pa, their EPSs, Ko, and/or its LPS for 20 h. Cytokine levels were assayed using ELISA kits. Data are shown as box plots (n = 6). *p < 0.05 vs Con. All were analyzed using unpaired *t* test.

In the in vitro study, *Klebsiella oxytoca* and its LPS induced IL-1β and IL-6 expression in macrophages, while treatment with *Enterococcus faecium, Pediococcus acidilactici,* or *Bifidobacterium longum* was not affected (Fig. 7). *Enterococcus faecium* and *Pediococcus acidilactici* synergistically increased IL-1β and IL-6 expression, while *Bifidobacterium longum* suppressed it. EPS of *Enterococcus faecium* or *Pediococcus acidilactici* singly did not induce IL-1β and IL-6 expression, while the combinations of EPSs with LPS synergistically increased these cytokine expression. However, *Bifidobacterium longum* EPS suppressed LPS-induced IL-1β and IL-6 expression.

## Discussion

Gut inflammation and dysbiosis are closely associated with the occurrence of psychiatric disorders^28,29^. In particular, the high prevalence of commensal gut Protoebacteria including *Enterobacteriaceae* is closely associated with IBD, including UC and CD, and overexpression of gut bacterial LPS^21,30^. The induction of depression by stressors such as immobilization and antibiotics triggers colitis and increases the gut Proteobacteria population and bacterial LPS production in mice^10,11^. The populations of *Alistipes* (Bacteroidetes) and *Enterobacteriacea*e are overpopulated in patients with depression^31^. However, to date, none of these species/strains were convincingly shown to be associated with the occurrence of IBD and depression.

In the present study, we found that IBD-F exhibited a significantly higher abundance of *Klebsiella oxytoca, Escherichia coli*, and *Enterococcus faecium* and a lower abundance of *Bifidobacterium longum* than HC-F. The populations of *Enterococcus faecium* and *Pediococcus acidilactici* were higher in IBD/D^+^-F vs. IBD/D^−^-F. Among these bacteria, *Klebsiella oxytoca* caused colitis and anxiety/depression in SPF mice most potently, followed by *Escherichia coli* and *Cronobacter sakazakii. Klebsiella oxytoca* yielded the highest level of mortality in SPF mice. Interestingly, these bacteria at a low dose caused anxiety with colitis, while depression with colitis was caused at a high dose. The occurrence of depression and colitis after exposure to *Klebsiella oxytoca* was more severe in germ-free mice than in specific pathogen-free mice: the occurrence of depression by *Klebsiella oxytoca* at a dose of 1 × 10^7^ CFU/mouse/day in germ-free mice was similar to that detected in specific-pathogen-free mice at a dose of 1 × 10^8^ CFU/mouse/day. However, *Enterococcus faecium, Pediococcus acidilactici*, and *Bifidobacterium longum* did not significantly cause colitis and anxiety/depression. Högenauer et al. reported that *Klebsiella oxytoca* may be a causative organism of antibiotic-associated hemorrhagic colitis^32^. Jang et al. reported that antibiotics-induced *Klebseilla oxytoca* and its lipopolysaccharide caused colitis and anxiety in mice^11^. Jang et al. also reported that IS-induced *Escherichia coli* and its lipopolysaccharide caused colitis and depression in mice^10^. These results suggest that the occurrence of anxiety/depression with colitis by *Enterobacteriaceae*, particularly *Klebsiella oxytoca, Escherichia coli,* or *Cronobacter sakazakii,* may be dependent on their titers overgrown in the gastrointestinal tract.

The combination of two bacteria among *Klebsiella oxytoca, Escherichia coli*, and *Cronobacter sakazakii* additively caused colitis and anxiety/depression. The combination of *Escherichia coli*, *Klebsiella oxytoca*, or *Cronobacter sakazakii* belonging to *Enterobacteriaceae* with *Pediococcus acidilactici* or *Enterococcus faecium* synergistically increased the occurrence of colitis and depression caused by parenteral bacteria of *Enterobacteriaceae*. However, *Pediococcus acidilactici* or *Enterococcus faecium* did not caused colitis and depression. Of these combinations, KoPa, and KoEf most strongly caused depression and colitis, followed by EcEf and EcPa. However, when combined with *Bifidobacterium longum*, which is also known as a probiotic^33^, it significantly suppressed the occurrence of depression and colitis caused by *Klebsiealla oxytoca,* or *Escherichia coli*. Khan et al. reported that the populations of Bifidobacteria and Lactobacilli were higher in the IBD-F than in the HC-F^34^. Barandouzi et al. reported that the population of *Bifidobacteriaceae*, but not *Bifidobacterium* sp., was higher in the gut microbiota of patients with depression than in those of HCs^35^. Cheung et al. reported that patients with depression exhibited a lower abundance of Bifidobacteria and a higher abundance of Proteobacteria including *Enterococcaceae* compared with those in the HCs^31^. Although some of *Pediococcus acidilactici* or *Enterococcus faecium* are well-known GRAS (generally recognized as safe) probiotics^36,37^, some of them are virulent pathogens, which produce cytolysis^38^. Cambronel et al. and Scardaci, et al. reported that probiotic *Enterococcus faecium* or *Enterococcus faecalis* increased catecholamine-dependently biofilm production, resulting in the increased adhesion to the intestinal cells^39,40^. These results suggest that gut bacteria belonging to *Enterobacteriaceae* together can additively cause colitis and anxiety/depression, *Enterococcus faecium* (*Enterococcaceae*) and *Pediococcus acidilactici* (*Lactobacillaceae*) can severely elevate the occurrence of colitis and depression by *Klebsiella oxytoca* or *Escherichia coli* (*Enterobacteriaceae*). However, *Bifidobacterium longum* (*Bifidobacteriaceae*) may suppress *Klebsiella oxytoca-* or *Escherichia coli-*inducible colitis and anxiety/depression. This suggestion is supported by the previous report that oral gavage of ampicillin/amoxicillin increases the populations of gut *Enterobacteriaceae* belonging to Proteobacteria and *Enterococcaceae*, which exhibits multi-antibiotic resistance^41^, in mice, resulting in the occurrence of colitis and anxiety.

The content of LPS was higher in the IBD-F than in the HC-F. LPS content was weakly, but not significantly, higher in the IBD/D^+^-F than in the IBD/D^−^-F. Oral gavage of *Klebsiealla oxytoca* and *Escherichia coli* increased the fecal and blood levels and hippocampal NF-κB^+^/Iba1^+^ and LPS^+^/Iba1^+^ cell populations in mice, while hippocampal and colonic tight-junction protein expression and hippocampal BDNF^+^/NeuN^+^ cell population decreased. Interestingly, mice highly detected LPS levels in the blood showed depression-like behaviors rather than anxiety-like behaviors. Vogelzangs et al. reported that LPS more sensitively stimulate anxiety than depression. These results suggest that LPS can cause anxiety/depression and anxiety may be processed to depression LPS-dependently^42^. Oral gavage of *Enterococcus faecium* and *Pediococcus acidilactici* did not affect blood and fecal LPS levels. However, the combined treatment of *Klebsiella oxytoca* or *Escherichia coli* with them increased blood and fecal LPS levels and hippocampal NF-κB^+^/Iba1^+^ and LPS^+^/Iba1^+^ cell populations. However, the simultaneous treatment of *Bifidobacterium longum* with *Klebsiella oxytoca* or *Escherichia coli* also reduced blood and fecal LPS level and hippocampal NF-κB^+^/Iba1^+^ and LPS^+^/Iba1^+^ cell populations in the hippocampus compared with those treated with *Klebsiella oxytoca* or *Escherichia coli* alone. Jang et al. reported that the overexpression of fecal LPS after exposure to *Escherichia coli* significantly suppressed the expression of tight-junction proteins in the brain and colon^11^. Kim et al. reported that *Escherichia coli* treatment increased the NF-κB^+^/Iba1^+^ and LPS^+^/Iba1^+^ cell populations in the hippocampus and LPS levels in the blood and feces and decreased the BDNF^+^/NeuN^+^ cell population and tight-junction protein expression^27^. Lee et al. also reported that oral gavage of *Escherichia coli* and its LPS caused colitis and neuroinflammation in mice^43^. The induction of gut dysbiosis by high-fat diet and ampicillin causes leaky gut in mice^11,44^. IBD patients suffer from leaky gut^45^, a condition that accelerates the translocation of gut bacteria and their by-products such as LPS across the intestinal mucosa into the blood and brain, resulting in neuroinflammation^46,47^. These results suggest that *Enterococcus faecium* and *Pediococcus acidilactici* can elevate the occurrence of depression by *Enterobacteriaceae* such as *Klebsiella oxytoca* and *Escherichia coli* through the regulation of gut microbiota LPS-stimulated NF-κB activation and BDNF expression.

Exposure to EPS, not CF, of *Enterococcus faecium* or *Pediococcus acidilactici* with *Klebsiella oxytoca* LPS synergistically increased the occurrence of colitis, neuroinflammation, and anxiety/depression in mice, while *Bifidobacterium longum* EPS suppressed them. Moreover, these EPSs of *Enterococcus faecium* and *Pediococcus acidilactici* elevated the translocation of orally gavaged fLPS into the brain, while *Bifidobacterium longum* EPS suppressed it. The simultaneous stimulation of their EPSs with *Klebsiella oxytoca* LPS synergistically increased neuroinflammation and colitis in mice and proinflammatory cytokine expression in macrophages in vitro. The cell wall EPSs of *Streptococcus* sp. (*Enterococcus* sp.) and *Bifidobacterium* sp. have extremely varied structures. EPSs of some Enterococcus sp. cause inflammation in animals structure-dependently^48^, while the EPSs of *Bifidobacteirum* sp. modulate immune responses including inflammation^49–51^. In particular, the EPS of *Bifidobacterium bifidum* enhances proinflammatory immune response or induces immunosuppressive regulatory T cells^52^. Moreover, some gram-positive and gram-negative exopolysaccharides cause chronic brain inflammation in animals^53^. These results suggest that *Enterococcus faecium, Pediococcus acidilactici,* and/or their EPSs may elevate the LPS production of gut bacteria such as *Klebsiella oxytoca* and *Escherichia coli,* cause colitis, which can increase the translocation of gut bacterial LPS into the brain, resulting in the increased occurrence of anxiety/depression with neuroinflammation. However, the EPS of *Bifidobacterium longum* may inhibit colitis and neuroinflammation by the regulation of NF-κB activation, leading to the attenuation of anxiety/depression. Nevertheless, the relationship between the structures of EPSs and the occurrence of anxiety/depression and between their structures and biological activities remains unclear.

In conclusion, the interaction between gut microbiota *Enterobacteriaceae, Enterococcaceae, Lactobacillaceae,* and *Bifidobacteriaceae* may control the occurrence of IBD, neuroinflammation, and anxiety/depression through the regulation of gut microbiota LPS production and LPS-induced NF-κB activation-mediated BDNF expression. *Enterococcus faecium* and *Pediococcus acidilactici* EPSs can elevate the occurrence of anxiety/depression with IBD by *Enterobacteriaceae*. In particular, the occurrence of depression may be dependent on the gut dysbiosis-attributable overproduction of bacterial LPS and EPS. The imbalanced overgrowth of *Enterococcus faecium* and *Pediococcus acidolactici,* known as a probiotic strain, and imbalanced overproduction of their EPSs may be a risk factor for the outbreak of anxiety/depression in patients with IBD.

## Methods

### Volunteers

Volunteers, consisting of HCs (average age, 38.2 ± 2.2 years) and patients with IBD/D^+^ (average age, 46.4 ± 15.3 years) and IBD/D^−^ (average age, 36.0 ± 12.6 years), were recruited from Kyung Hee University (Seoul, Korea) (Supplementary Table S1), as previously reported^25^. All patients with IBD enrolled in the study were > 13 years of age at the diagnosis of IBD, and all diagnoses were confirmed by previously established international criteria based on clinical, endoscopic, histopathological, and radiological findings^54^. The study protocol and informed consent forms for the stool collection were approved by the Committee for the Care and Use of Clinical Study of the Medical School of Kyung Hee University (IRB File No., KHUH 2018-03-006-018 and KHUH 2018-12-004-003). In a patient aged under 18, informed consent was obtained from a parent. All experiments were performed in accordance with relevant named guidelines regulations. We confirmed that informed consent was obtained from all participants before the initiation of the study and that all methods involving human participants were carried out in accordance with the ethical principles of the Declaration of Helsinki and the Korean Good Clinical Practice guidelines.

### Animals

SPF C57BL/6 mice (male, 6 weeks old, 19–21 g) were purchased from Koatech Inc. (Seoul, Korea). Mice were kept in wire cages under a ventilated condition (20–22 °C, 50% ± 10% humidity, and 12-h/12-h light/dark cycle) and fed standard laboratory chow and water ad libitum. Germ-free C57BL/6 J mice (male, 18–21 g, 5 weeks old) were purchased from Clea Japan Inc. (Tokyo, Japan). Germ-free mice were housed in flexible film plastic isolators. All conditions were kept sterile in accordance with The Guidelines for Laboratory Germ-free Animals Care and Usage. Mice were used in the experiments after acclimation for 1 week. All animal experiments were approved by the Institutional Animal Care and Use Committee of Kyung Hee University [IACUC No., KUASP(SE)-18-033, KUASP(SE)-19-290, and KUASP(SE)-20-078] and were performed according to the NIH and University Guide for Laboratory Animals Care and Usage. This study additionally adheres to standards articulated in the ARRIVE guidelines.

### Culture of gut bacteria

The fresh feces (0.5 g) of IBD patients and HCs were immediately collected, immediately suspended in 4.5 mL of general anaerobic medium (GAM, Nissui Pharmaceutical Inc., Tokyo, Japan) broth on ice, inoculated onto BL and DHL agar plates (Nissui Pharmaceutical Co. Ltd.), and cultured aerobically (for DHL agar plates) at 37 °C for 1 day or anaerobically (for BL and mEn agar plates) at 37 °C for 3 days^27,43^. The colonies grown in agar plates were inoculated into GAM semi-solid media. These bacteria were identified using Gram staining, 16S rRNA gene sequencing, and API kits, as previously reported^10,21^. For in vitro and in vivo experiments, gut bacteria were anaerobically cultured in the GAM broth at 37 °C (0.8–1.0 at 600 nm)^10,21,42^. Cultured bacteria were collected by centrifugation for 20 min at 5000*g*, and washed twice with saline. The collected cells (1 × 10^10^ CFU/mL) were suspended in saline.

### Isolation of LPS and EPS

*Pediococcus acidilactici, Enterococcus faecium* and *Bifidobacterium longum* anaerobically cultured in the GAM broth at 37 °C were collected, washed with distilled water, suspended in distilled water, and sonicated according to the methods of Kim and Kobashi^55^. The sonicated suspension was centrifuged (10,000*g*, 4 ℃, 30 min). The supernatant faction was freeze-dried and used as cytosolic fraction (CF). EPSs were purified from their precipitates according to the method of Smitinont et al.^56^ *Klebsiella oxytoca* was anaerobically cultured in the GAM broth at 37 °C, and centrifuged for 20 min at 5000 g, and washed twice with saline. The collected cells (1 × 10^10^ CFU/mL) were suspended in distilled water. Its LPS was purified using a LPS extraction kit (iNtRON Biotechnolgy, Seongnam, Republic of Korea)^42^. FITC (F7250 Sigma, Aldrich)-conjugated LPS (FITC to LPS ratio, 0.2) was prepared as described by Park et al.^57^.

### Culture of macrophage cells

Macrophages were isolated from the peritoneal cavity of mice intraperitoneally injected with sodium thioglycolate according to the method of Kim et al.^27^ Isolated macrophages were suspended in RPMI 1640 containing 10% fetal bovine serum and 1% antibiotics (RFA), seeded in 6-well plate, incubated at 37 °C for a day, and washed with RFA. The macrophages were treated in the presence or absence of test agents for 20 h. Cytokine levels were assayed using ELISA kits.

### Treatment with gut bacteria in mice

To investigate whether gut microbiota could cause depression and/or colitis, we orally gavaged gut bacteria (1 × 10^6^, 1 × 10^7^, 1 × 10^8^ or 1 × 10^9^ CFU/mouse/day, suspended singly or together in saline) in mice once a day for 5 days and measured anxiety/depression-like behaviors for 3 day (Supplementary Fig. S9a,b).

To investigate whether the components of gut microbiota such as EPS, LPS, and CF could cause depression and colitis, *Klebsiella oxytoca* LPS (10 μg/kg/day for i.p. or 20 mg/kg/day for p.o.) in the absence or presence of EPG or CF from *Pediococcus acidilactici, Enterococcus faecium,* or *Bifidobacterium longum* (10 μg/kg/day for i.p. or 20 mg/kg/day for p.o.) were orally gavaged or intraperitoneally injected in mice once a day for 5 days (Supplementary Fig. S9c).

FITC-conjugated LPS (20 mg/kg/day) was orally gavaged daily for 2 days from 24 h after EPS or LPS conjugated without FITC was gavaged for 3 days. Anxiety-/depression-like behaviors were measured in the EPM and MB tasks, TST, and FST on the next day after EPS or LPS was treated. Mice were sacrificed 12 h after the final behavioral tasks. Colons and brains were removed and stored at – 80 °C for ELISA and immunoblotting.

### Behavioral tasks

The EPM task was performed in a plus-maze apparatus (consisting of two open [30 × 7 cm] and two enclosed [30 × 7 cm] arm with 20-cm-high walls extending from a central platform [7 × 7 cm]), according to the method of Jang et al.^10^ MB task was assessed in a smooth, opaque plastic cage (30 × 35 × 13 cm) with a 5 cm layer of sawdust and 30 marbles on top of it (five rows or marbles regularly spaced at 2 cm away from the walls) according to Jang et al.^10^. The number of marbles buried for 30 min was counted. The TST was performed on the edge of a table, at 30 cm above it, for 5 min according to the method of Kim et al.^27^. Mice were judged to be immobile, when they did not move and hung passively. The FST was performed in a round transparent plastic jar (20 × 40 cm^3^) containing fresh water (25 °C) to a height of 25 cm for 5 min, according to Kim et al.^27^.

### Immunofluorescence assay

The brains and colons were removed from mice perfused and post-fixed with paraformaldehyde, cytoprotected in 30% sucrose solution and cryosectioned. Sectioned tissues were immunostained according to the method of Lee et al.^43^.

### Enzyme-linked immunosorbent assay (ELISA) and immunoblotting

Colon and brain tisusses were homogenized in the RIPA lysis buffer containing 1% phosphatase inhibitor cocktail and 1% protease inhibitor cocktail (RPP) on ice and centrifuged at 15,000×*g* at 4 °C for 15 min. The levels of cytokines in brain and colon tissues and corticosterone and cytokines in the plasma were assayed according to the method of Kim et al.^27^.

For the immunoblotting, the supernatants of the colon and cultured cell homogenates were subjected to sodium dodecyl sulfate–polyacrylamide gel electrophoresis and transferred to nitrocellulose membrane^27^. Proteins were probed with antibodies, detected with horseradish peroxidase-conjugated secondary antibodies, and visualized with ECL detection kit.

### Myeloperoxidase activity and limulus amebocyte lysate (LAL) assay

Myeloperoxidase activity was assayed according to the method of Jang et al.^10^. Blood and fecal endotoxin levels were assayed using an LAL assay kit (Cape Cod Inc., E. Falmouth, MA) according to the method of Jang et al.^10^.

### Microbiota composition analysis

Bacterial genomic DNAs were extracted for the fresh feces of HCs, IBD patients, and feces-treated mice using a QIAamp DNA stool mini kit (Qiagen according to the method of Kim et al.^27^. Amplification of genomic DNA was performed using barcoded primers targeted the bacterial 16S rRNA V4 region gene and sequenced using Illumina iSeq 100 (San Diego, CA). Sequencing reads were deposited in the short read archive of NCBI under accession number PRJNA666980.

### Quantitative real-time polymerase chain reaction (qPCR)

qPCR for gut bacteria was performed on the Rotor-Gene Q® thermocycler using DNA polymerase and SYBR Green I (Takara Bio Inc.: RR820A)^27^. PCR amplification reaction was carried out as follows: initial denaturation at 95 °C for 30 s, followed by 45 cycles of denaturation at 95 °C for 5 s, annealing at 55 °C for 30 min, and extension at 72 °C for 30 s^27^. Primers for qPCR are indicated in Supplementary Table S2.

### Statistics

Experimental data are described as the mean ± SD using GraphPad Prism 8 (GraphPad Software, Inc., San Diego, CA, USA). Significant differences were analyzed using non-parametric ANOVA with Kruskal–Wallis test, one-way ANOVA with post-hoc Bonferroni's comparisons test, or unpaired t test, (p < 0.05).

## Supplementary Information

Supplementary Information.
