# Author Correction: *Enterococcus faecium* and *Pediococcus acidilactici* deteriorate *Enterobacteriaceae*-induced depression and colitis in mice

**DOI:** 10.1038/s41598-023-39969-8

**Published:** 2023-08-10

**Authors:** Hyo-Min Jang, Jeon-Kyung Kim, Min-Kyung Joo, Yoon-Jung Shin, Kyung-Eon Lee, Chang Kyun Lee, Hyo-Jong Kim, Dong-Hyun Kim

**Affiliations:** 1 Neurobiota Research Center, College of Pharmacy, Kyung Hee University, 26, Kyungheedae-ro, Dongdaemun-gu, Seoul, 02447 Korea; 2 College of Pharmacy, Jeonbuk National University, 26, Jeonju, 54896 Korea; 3 Department of Internal Medicine, Kyung Hee University School of Medicine, Seoul, 02447 Korea

Correction to: *Scientific Reports* https://doi.org/10.1038/s41598-022-13629-9, published online 07 June 2022

The original version of this Article contained an error in Figure 3, panel c, where the LPS^+^/Iba1^+^ and NF-κB^+^/Iba1^+^ images were duplicated. The original Figure 3 and accompanying legend appear below.

**Figure 3:**
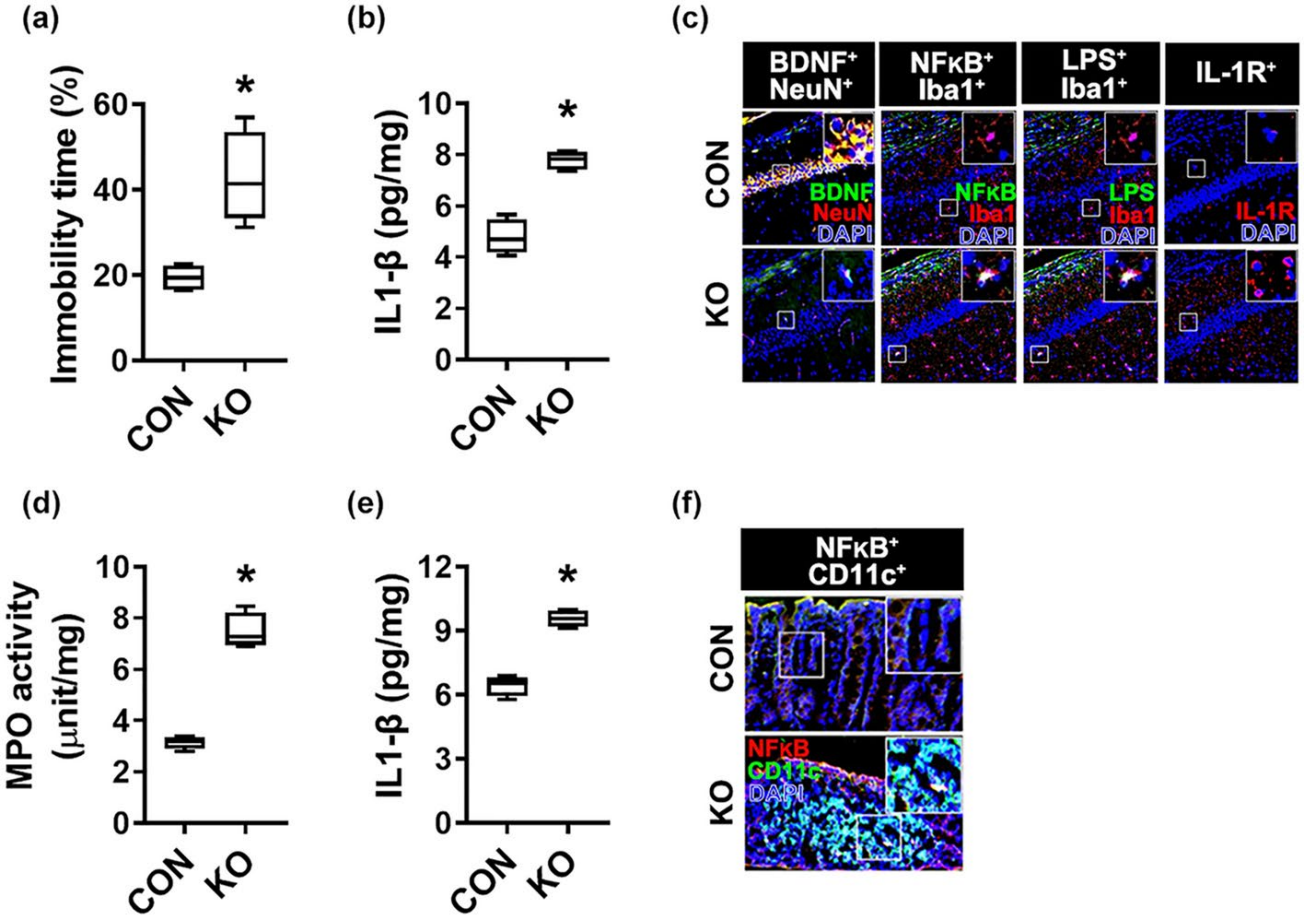
Effect of *Klebsiella oxytoca* on the occurrence of depression and colitis in germ-free mice. Effect on the occurrence of depression-like behaviors (**a**) and hippocampal IL-1β level (**b**), BDNF^+^/NeuN^+^ (**c**), NF-κB^+^/Iba1^+^ (**d**), LPS^+^/Iba1^+^ (**e**), and IL-1R^+^ cell populations (**f**) in germ-free mice. *Klebsiella oxytoca* (KO, 1 × 10^7^ CFU/mouse/day) were orally gavaged for 5 days in mice (n = 6, in specific-germ-free mice; n = 4, in germ-free mice). Control mice (NC) were treated with vehicle (saline) instead of the bacterial suspension. Data are shown as box plots. Means with same letters are not significantly different (p < 0.05). All were analyzed using unpaired t-test.

The original Article has been corrected.

