# Supplementary material for: Enterococcus faecium and Pediococcus acidilactici deteriorate Enterobacteriaceae-induced depression and colitis in mice
Source: Sci Rep. 2022 Jun 7;12:9389. doi: 10.1038/s41598-022-13629-9 (PMC9174183; doi:10.1038/s41598-022-13629-9)
Supplement: Supplementary file 1 — Supplementary Information. [file 41598_2022_13629_MOESM1_ESM.pdf]

[Supplementary materials]

***Enterococcus faecium* and *Pediococcus acidilactici* deteriorate *Enterobacteriaceae*-induced depression and colitis in mice**

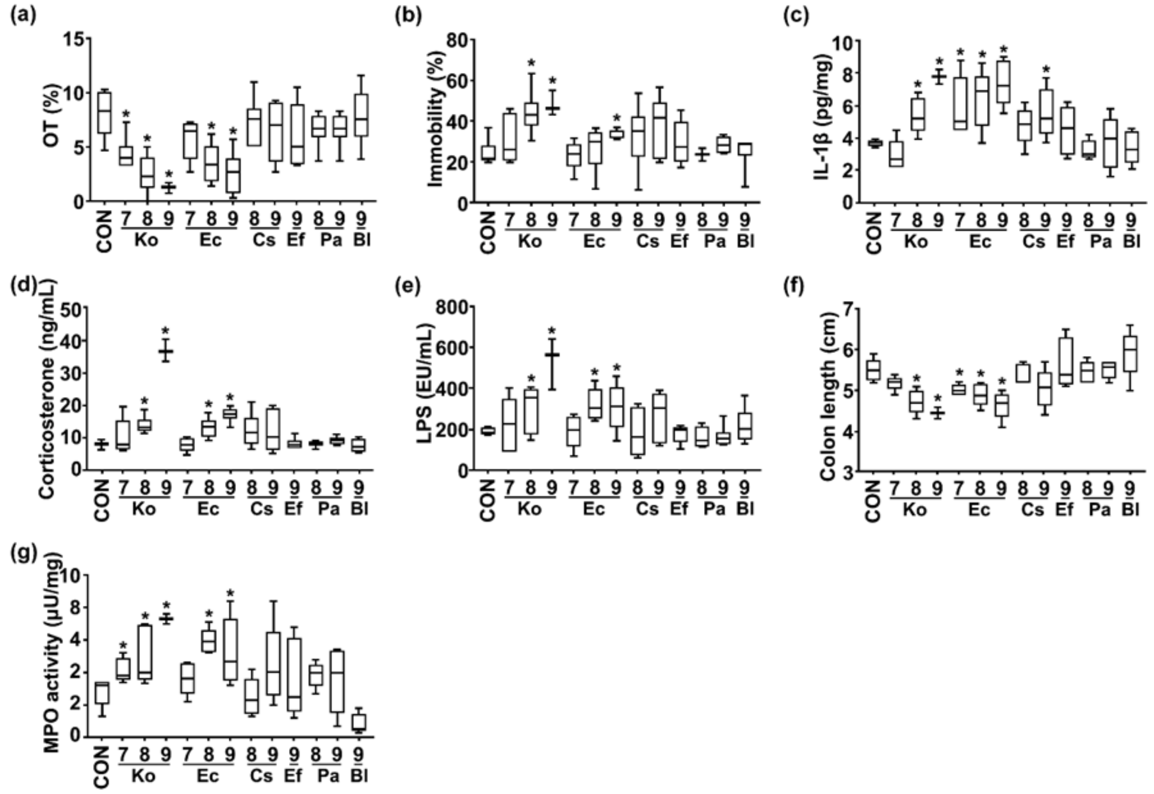

Figure S1. Effect of gut bacteria on the occurrence of anxiety/depression and colitis in mice. Effects on the time spent in open arms (OT) in the EPM task (a) and immobility time in the TST (b). Effects on the IL-1β expression in the hippocampus (c). Effects on the corticosterone (d) and LPS levels (e) in the blood. Effects on the colon length (f) and myeloperoxidase activity (g) in the colon. *Klebsiella oxytoca* (Ko), *Escherichia coli* (Ec), *Cronobacter sakazakii* (Cs), *Enterococcus faecium* (Ef), *Pediococcus acidilactici* (Pa), or *Bifidobacterium longum* (Bl) at doses of 1×10^6 [6], 1×10^7 [7], 1×10^8 [8], 1×10^9 [9] CFU/mouse/day) was orally gavaged in mice once a day for 5 days. Control mice (Con) were treated with vehicle (saline) instead of the gut bacterial suspension. Data are shown as box plots (n=6). $^*p<0.05$ vs Con. Means with same letters are not significantly different ($p < 0.05$). All were analyzed using unpaired *t* test.

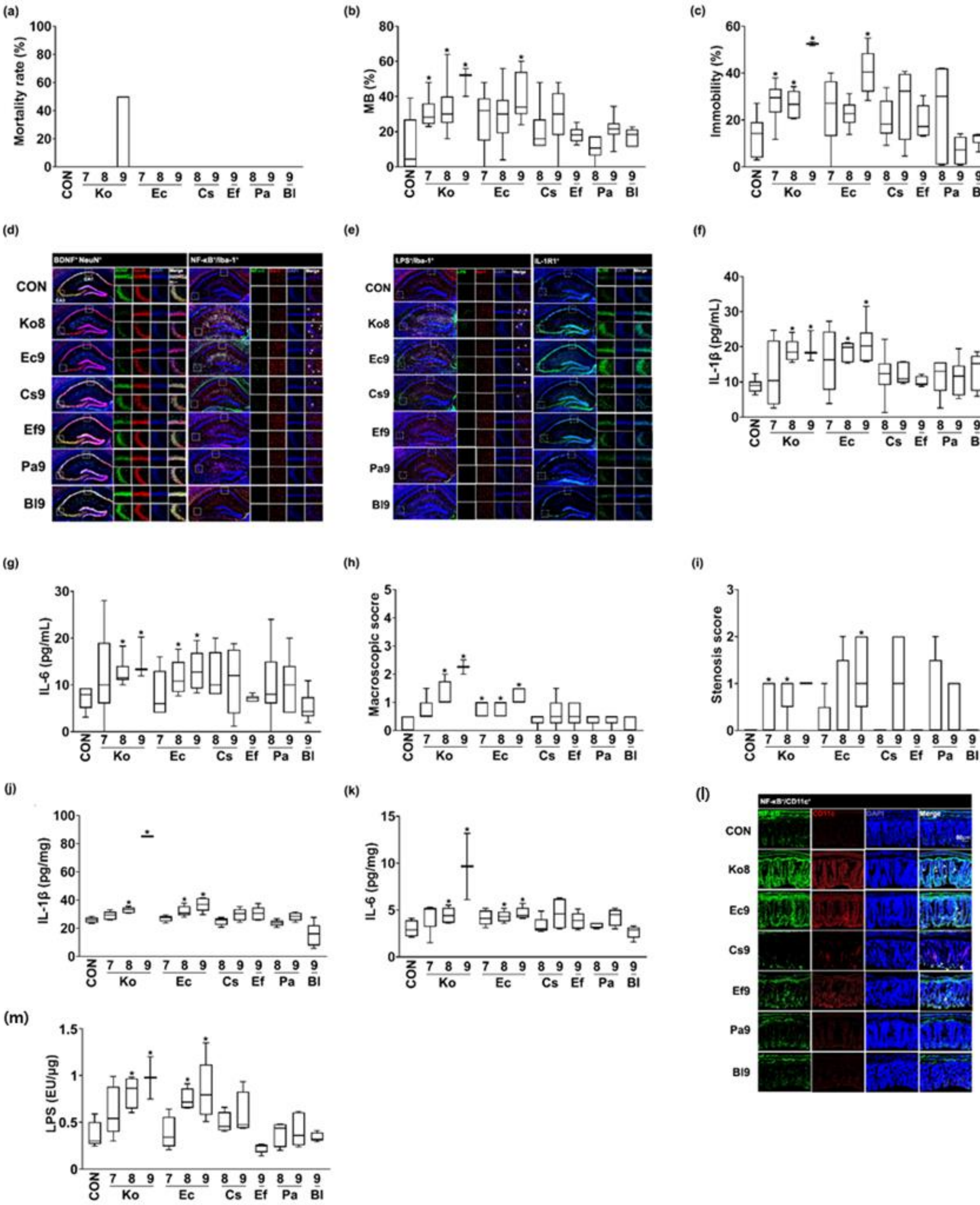

Figure S2. Effect of *Klebsiella oxytoca* (Ko)*, Escherichia coli* (Ec), *Cronobacter sakazakii* (Cs), *Enterococcus faecium* (Ef)*, Pediococcus acidilactici* (Pa), or *Bifidobacterium longum* (Bl) on the occurrence of anxiety/depression and colitis in mice. (a) Effects on the mortality. Effects on the occurrence of anxiety/depression in the MB task (b), and FST (c). Effects on the BDNF$^+$/NeuN$^+$ and NF-κB+/Iba-1$^+$ cell populations (d) and LPS$^+$/Iba1$^+$ and IL-1R$^+$ cell populations (e) in the hippocampus. Effects on the IL-1β (f) and IL-6 expression (g) in the blood. Effects on macroscopic score (h), stenosis score (i), IL-1β (j), and IL-6 (k), and NF-κB$^+$/CD11c$^+$ cell population (l) in the colon. (m) Effects in the fecal LPS level. Each bacterial suspension at doses of 1×10^6 [6], 1×10^7 [7], 1×10^8 [8], 1×10^9 [9] CFU/mouse/day) was orally gavaged in six mice once a day for 5 days. Control mice (Con) were treated with vehicle (saline) instead of gut bacterial suspension. Data are shown as box plots (n=6). $^*p<0.05$ vs

Con. P Means with same letters are not significantly different ($p < 0.05$). All was analyzed by using unpaired *t* test.

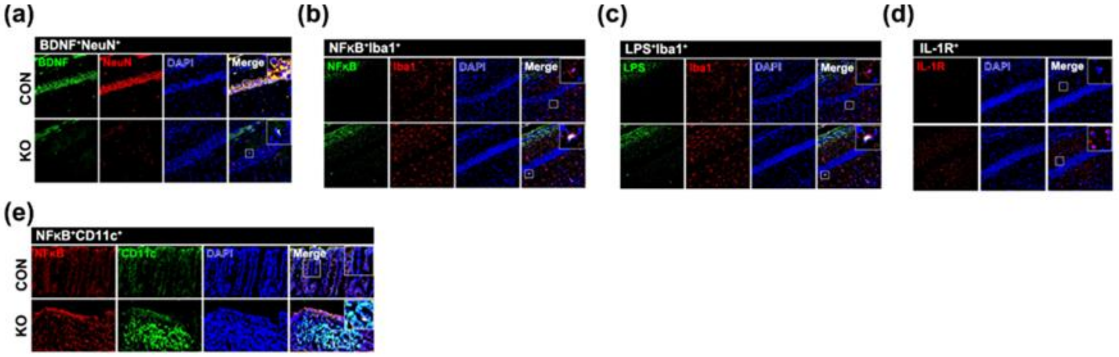

Figure S3. Effect of *Klebsiella oxytoca* on the occurrence of depression and colitis in germ-free mice. Effect on hippocampal BDNF+/NeuN+ (a), NF-κB+/Iba1+ (b), LPS+/Iba1+ (d), and IL-1R+ cell populations (d) and colonic NF-κB+/CD11c+ cell population in germ-free mice. *Klebsiella oxytoca* (KO, 1×10^7 CFU/mouse/day) were orally gavaged for 5 days in mice (n=4). Control mice (NC) were treated with vehicle (saline) instead of the bacterial suspension.

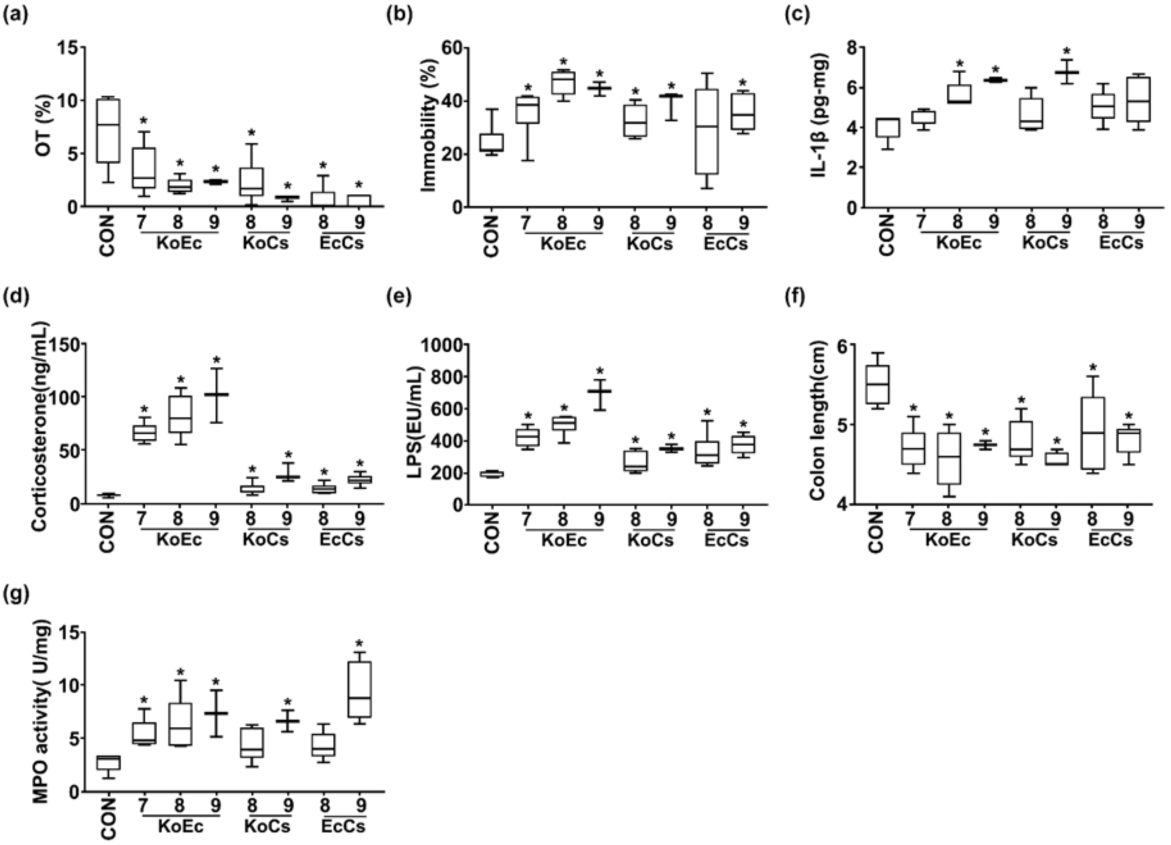

Figure S4. Combined effects of two bacteria belonging to *Enterobacteriaceae* on the occurrence of anxiety/depression and colitis in mice. Effects on the occurrence of anxiety/depression in the EPM task (a) and TST (b). Effects on the IL-1β expression in the hippocampus (c). Effects on the corticosterone (d) and LPS levels (e) in the blood. Effects on the colon length (f) and myeloperoxidase activity (g) in the colon. Among *Klebsiella oxytoca*

(Ko), *Escherichia coli* (Ec), and *Cronobacter sakazakii* (Cs), two bacterial (KoEc, 1:1 of KO and Ec; KoCs, 1:1 of Ko and Cs; EcCS, 1:1 of Ec and Cs) combinations at doses of 1×10^6 [6], 1×10^7 [7], 1×10^8 [8], 1×10^9 [9] CFU/mouse/day) were orally gavaged once a day for 5 days in mice. Control mice were treated with vehicle (saline) instead of gut bacterial suspension. Data are shown as box plots (n=6). $^*p<0.05$ vs Con. All were analyzed using unpaired *t* test.

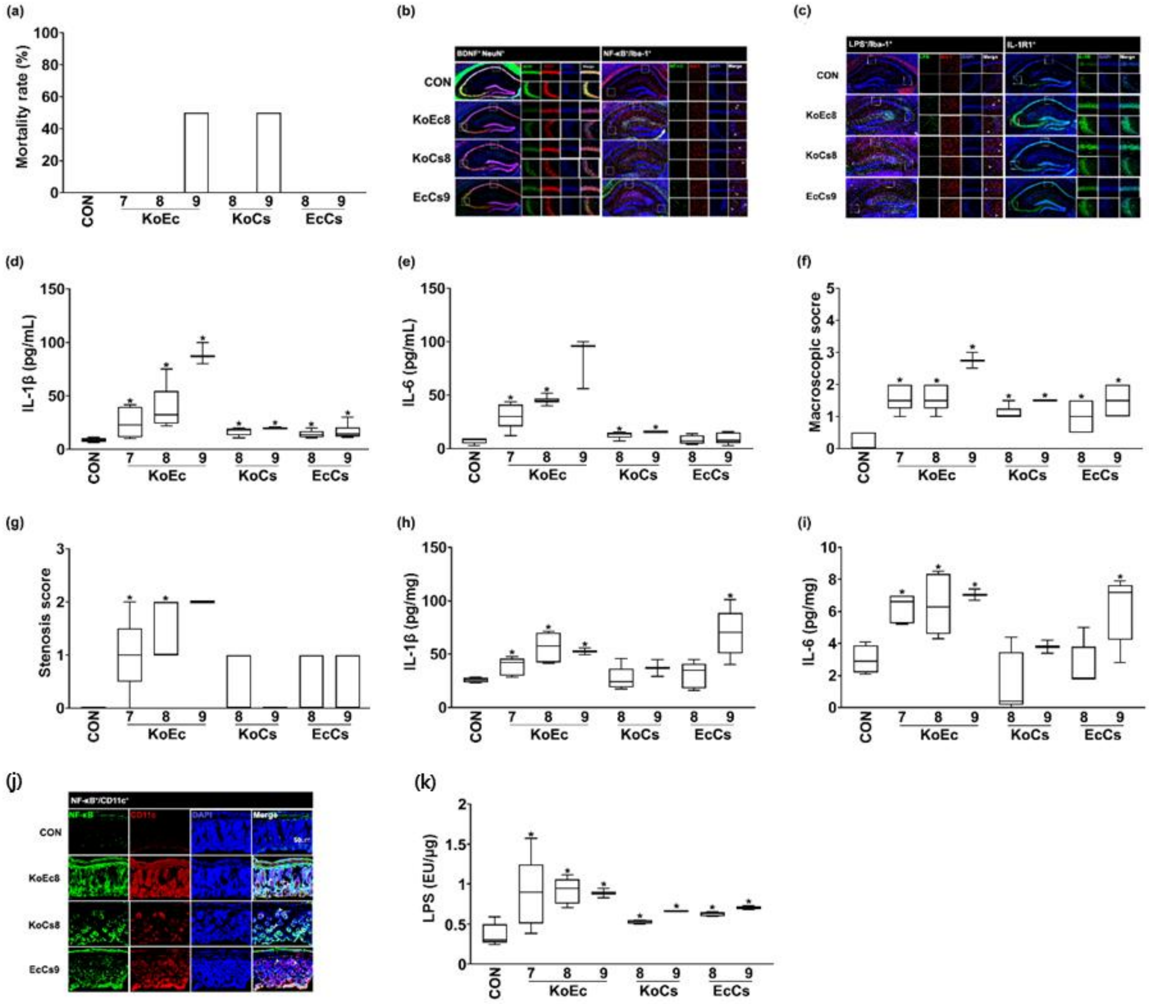

Figure S5. Effects of two bacterial combinations among *Klebsiella oxytoca* (Ko), *Escherichia coli* (Ec), and *Cronobacter sakazakii* (Cs) on the occurrence of anxiety/depression and colitis in mice. (a) Effects on the mortality. Effects on the BDNF$^+$/NeuN$^+$ and NF-κB+/Iba-1$^+$ cell populations (b) and LPS$^+$/Iba1$^+$ and IL-1R$^+$ cell populations (c) in the hippocampus. Effects on the IL-1β (d) and IL-6 expression (e) in the blood. Effects on macroscopic score (f), stenosis score (g), IL-1β (h), IL-6 (i), and NF-κB$^+$/CD11c$^+$ cell population (j) in the colon. (k) Effects in the fecal LPS level. Each two bacteria (KoEc, 1:1 of KO and Ec; KoCs, 1:1 of Ko and Cs; EcCS, 1:1 of Ec and Cs) combination at doses of 1×10^6 [6], 1×10^7 [7], 1×10^8 [8], 1×10^9 [9] CFU/mouse/day) was orally gavaged in six mice once a day for 5 days. Control mice were treated with vehicle (saline) instead of gut bacterial suspension. Data are shown as box plots (n=6). $^*p<0.05$ vs Con. All was analyzed by using unpaired *t* test.

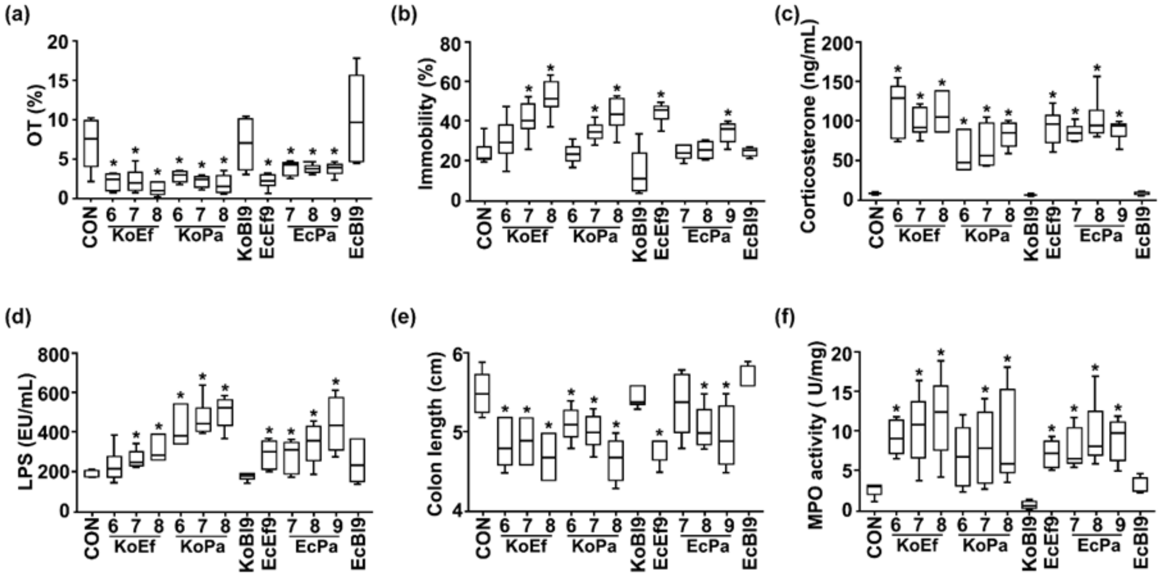

Figure S6. Combined effects of *Enterococcus faecium* (Ef)*, Pediococcus acidilactici* (Pa), or *Bifidobacterium longum* (Bl) with *Enterobacteriaceae* on the occurrence of anxiety/depression and colitis in mice. Effects on the occurrence of anxiety/depression in the EPM task (a) and TST (b). Effects on the IL-1β expression in the hippocampus (c). (d) Effects on the corticosterone in the blood. Effects on the colon length (e) and myeloperoxidase activity (f) in the colon. Each two bacteria (KoEf, 1:1 of Ko and Ef; KoPa, 1:1 of Ko and Pa; KoBl, 1:1 of Ko and Bl; EcEf, 1:1 of Ec and Ef; EcPa, 1:1 of Ec and Pa; EcBl, 1:1 of Ec and Bl) combination at doses of 1×10^6 [6], 1×10^7 [7], 1×10^8 [8], 1×10^9 [9] CFU/mouse/day) was orally gavaged once a day for 5 days in mice. Control mice were treated with vehicle (saline) instead of gut bacterial suspension. Data are shown as box plots (n=8). $^*p<0.05$ vs Con. All were analyzed using unpaired *t* test.

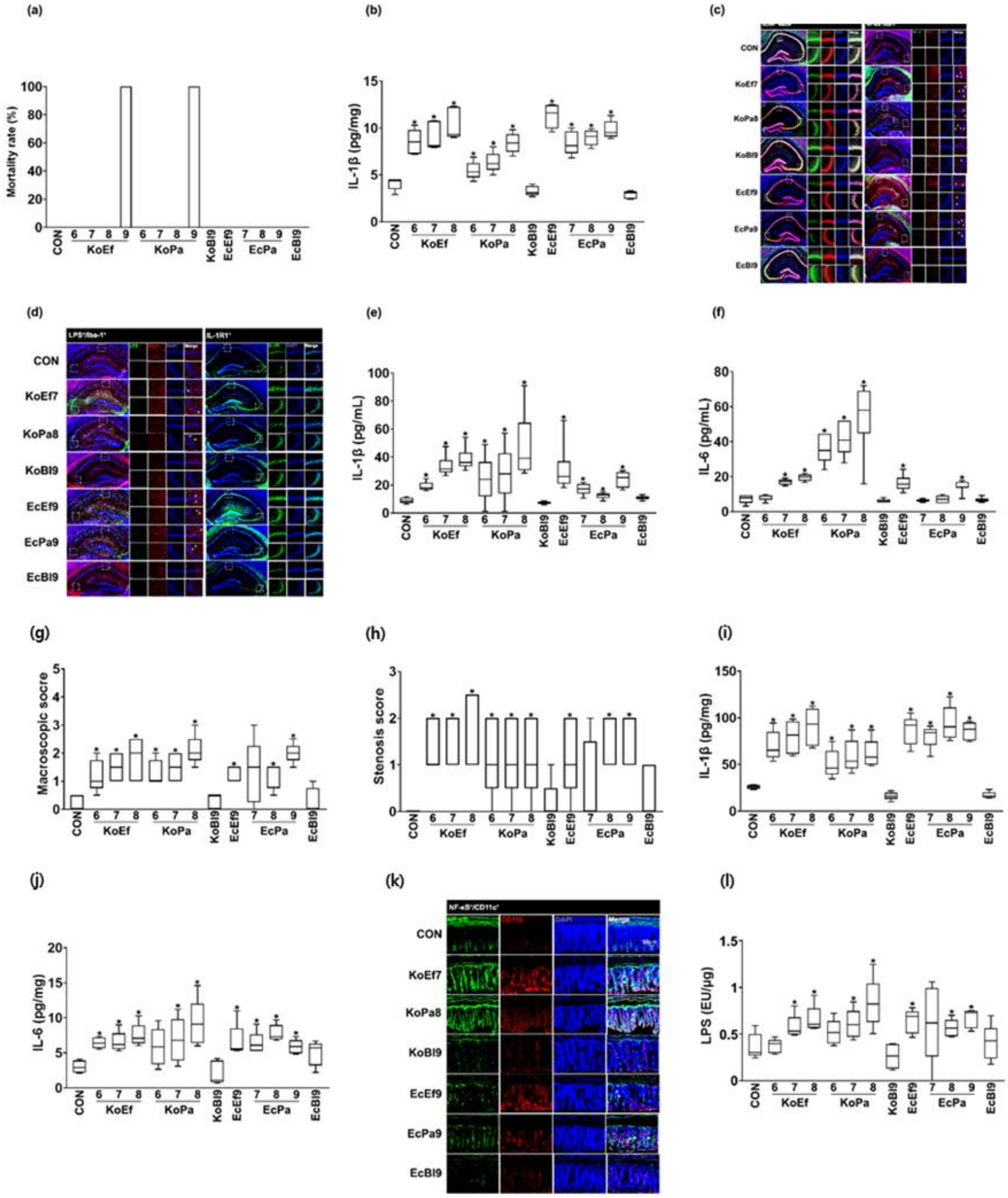

Figure S7. Combined effects of *Enterococcus faecium* (Ef)*, Pediococcus acidilactici* (Pa), or *Bifidobacterium longum* (Bl) with *Enterobacteriaceae* (*Klebsiella oxytoca* [Ko] or *Escherichia coli* [Ec]) on the occurrence of anxiety/depression and colitis in mice. (a) Effects on the mortality. Effects on the IL-1β expression (b), BDNF$^+$/NeuN$^+$ and NF-κB+/Iba-1$^+$ cell populations (c), and LPS$^+$/Iba1$^+$ and IL-1R$^+$ cell populations (d) in the hippocampus. Effects on the IL-1β (e) and IL-6 expression (f) in the blood. Effects on macroscopic score (g), stenosis score (h), IL-1β (i), and IL-6 expression (j), and NF-κB$^+$/CD11c$^+$ cell population (k) in the colon. (l) Effects in the fecal LPS level. Each two bacteria (KoEf, 1:1 of Ko and Ef; KoPa, 1:1 of Ko and Pa; KoBl, 1:1 of Ko and Bl; EcEf, 1:1 of Ec and Ef; EcPa, 1:1 of Ec and Pa; EcBl, 1:1 of Ec and Bl) combination at doses of 1×10^6 [6], 1×10^7 [7], 1×10^8 [8], 1×10^9 [9]

CFU/mouse/day) was orally gavaged in six mice once a day for 5 days. Control mice were treated with vehicle (saline) instead of gut bacterial suspension. Data are shown as box plots (n=8). *p<0.05 vs Con. All was analyzed by using unpaired *t* test.

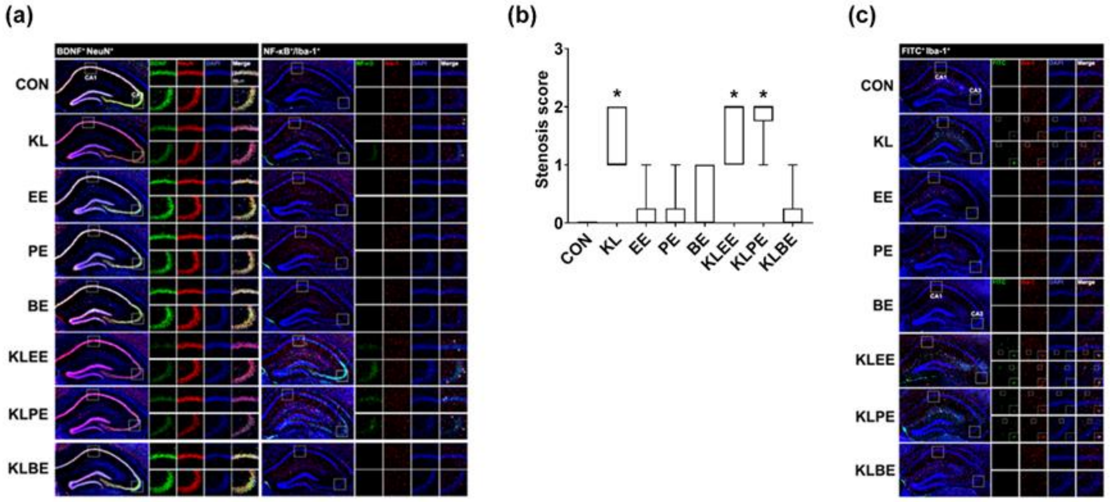

Figure S8. Effects of orally gavaged EPS and LPS on the occurrence of anxiety/depression and colitis in mice. Effects on the BDNF$^+$/NeuN$^+$ and NF-κB+/Iba-1$^+$ cell populations in the hippocampus (a) and stenosis score (b) in the colon. (c) Effects of EPSs on the translocation of FITC-conjugated LPS into the hippocampus. Test agents (CON, vehicle [saline]; EE, ng/kg of *Enterococcus faecium* exopolysaccharide; PE, ng/kg of *Pediococcus acidilactici* exopolysaccharide; BE, ng/kg of *Bifidobacterium longum* exopolysaccharide; KL, ng/kg of of *Klebsiella oxytoca* lipoplysaccharide; KLEE, of Ko and Ef (1:1); KLPE, of Ko and Pa (1:1); KLBE, of Ko and Bl (1:1) were orally gavaged in six mice once a day for 5 days. FITC-conjugated LPS was orally gavagaed once for 2 days after oral gavage for EPS or LPS not conjugated with FITC. Control mice were treated with vehicle (saline) instead of gut bacterial suspension. Data are shown as box plots (n=8). *p<0.05 vs Con. All was analyzed by using unpaired *t* test.

(a)

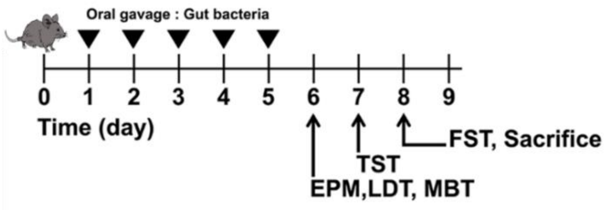

(b)

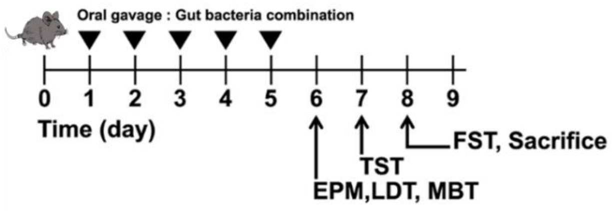

(c)

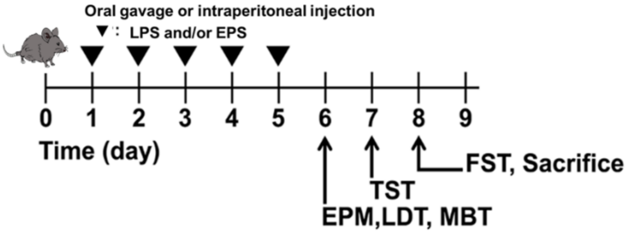

Figure S9. Experimental schedules of mice. (a) Schedule of gut bacteria treatment in mice. (b) Schedule of combined bacteria treatment in mice. (c) Schedule of combined treatment with LPS and/or EPS

Table S1. Clinical characteristics of study participants

Group		Age (year of birth)	Height (m)	Weight (kg)	Body mass index[1)]	Psychiatric disorders[2)]		Medication[3)]
						HADS-A	HADS-D	
Healthy control (HC)		39 (1979)	1.65	53.6	19.7	N/A	N/A	N/A
		37 (1982)	1.48	58.6	26.7	N/A	N/A	N/A
		41 (1978)	1.60	52.6	20.6	N/A	N/A	N/A
		36 (1938)	1.58	54	21.6	N/A	N/A	N/A
Mean±SD		38.2±2.2	1.6±0.1	54.7±2.7	22.2±3.1	-	-	-
IBD/D⁻ [4)]	UC	42 (1977)	1.61	46.6	18.0	4	5	B
		34 (1984)	1.74	79	26.1	2	6	B
		51 (1967)	1.8	79	24.4	4	5	B
		48 (1971)	1.78	76	24.0	9	5	None
		44 (1976)	1.75	73	23.8	8	6	B,C
	CD	30 (1990)	1.68	52	18.4	2	3	A,B
		14 (2006)	1.62	55	21.0	3	2	C
		25 (1994)	1.77	65	20.7	5	4	B,C
Mean±SD		36.0±12.6	1.70±0.1	65.7±13.0	22.1±3.0	4.6±2.6	4.5±1.4	-
IBD/D⁺ [5)]	UC	59 (1959)	1.73	60	20.0	15	16	None
		57 (1962)	1.58	53	21.2	9	13	B
		64 (1955)	1.68	60	21.3	11	11	C
		26 (1993)	1.62	56	21.3	19	11	A,B
	CD	26 (1992)	1.68	58	20.5	19	14	C
		46 (1972)	1.77	57	18.2	7	14	B,C
		47 (1971)	1.68	60	21.3	4	15	C
Mean±SD		46.4±15.3	1.70±0.1	57.7±2.6	20.5±1.2	12.0±5.9	13.4±1.9	-

1) Body mass index (BMI) BMI is defined as the body mass divided by the square of the body

height, and is universally expressed in units of kg/m^2, resulting from mass in kilograms and height in meters.

[2] Psychiatric disorders were described as HADS (Hospital anxiety and depression scale) A (anxiety) and D (depression) score.

[3] In the case of the healthy control group, it was investigated whether medicines that can affect the gut microbiota, such as analgesics, anti-inflammatory drugs, and antibiotics, were administered within 3 months. And in the case of patients, medications were expressed in A (Steroid), B (Immuno-modulators, such as azathioprine or methotrexate) and C (Biologics/small molecules) for therapeutic purpose.

[4] Inflammatory bowel disease patients without depression (score, <10 on HADS-D)

[5] Inflammatory bowel disease patients with depression (score, >10 on HADS-D)

Table S2. Primers for the qPCR of gut bacteria

	Primer	
	Forward	Reverse
Klebsiella sp.	5'-AGAGTTTGATCCTGGCTCAG-3'	5'-GGTTACCTTGTTACGACTT-3'
Klebsiella sp. oxytoca	5'-ACCTTACCTACTCTTGACATCC-3'	5'-CCCACCTTCCTCCAGTTTATC-3'
Escherichia coli	5'-CAGCCACACTGGAACTGAGA-3'	5'-GTTAGCCGGTGCTTCTTCTG-3'
Cronobacter sakazakii	5'-GTAGCTAATACCGCATAACGTC-3'	5'-AACCACAACACCTTCCTC-3'
Pedioccus acidilactici	5'-ACGCATTAAGTAATCCGCC-3'	5'-ACCACCTGTCATTCTGTCC-3'
Enterococcus faecium	5'-CGCATGGTTTTGATTTGAAAGG-3'	5'-TGTCTCAGTCCCAATGTGG-3'
Bifidobacterium longum	5'-CGCGTCYGGTGTGAAAG-3'	5'-CCCCACATCCAGCATCCA-3'
16s rRNA	5'-TCGTCGGCAGCGTCAGATGTGTATAAGAGACAGGTGCCAGCMGCCGCGGTAA-3'	5'-GTCTCGTGGGCTCGGAGATGTGTATAAGAGACAGGGACTACHV GGGTWTCTAAT-3'
